# Comprehensive Transcriptome Analysis of Developing Xylem Responding to Artificial Bending and Gravitational Stimuli in *Betula platyphylla*

**DOI:** 10.1371/journal.pone.0087566

**Published:** 2014-02-20

**Authors:** Chao Wang, Nan Zhang, Caiqiu Gao, Zhiyuan Cui, Dan Sun, Chuanping Yang, Yucheng Wang

**Affiliations:** 1 State Key Laboratory of Tree Genetics and Breeding (Northeast Forestry University), Harbin, China; Nazarbayev University, Kazakhstan

## Abstract

*Betula platyphylla Suk* (birch) is a fast-growing woody species that is important in pulp industries and the biofuels. However, as an important pulp species, few studies had been performed on its wood formation. In the present study, we investigated the molecular responses of birch xylem to artificial bending and gravitational stimuli. After trunks of birch trees were subjected to bending for 8 weeks, the cellulose content was significantly greater in tension wood (TW) than in opposite wood (OW) or normal wood (NW), whereas the lignin content in TW was significantly lower than that in OW and NW. In addition, TW grew more rapidly than OW and generated TW-specific fibers with an additional G-layer. Three transcriptome libraries were constructed from TW, OW and NW of *B. platyphylla*, respectively, after the plants were subjected to artificial bending. Overall, 80,909 nonredundant unigenes with a mean size of 768 nt were assembled. Expression profiles were generated, and 9,684 genes were found to be significantly differentially expressed among the TW, OW and NW libraries. These included genes involved in secondary cell wall structure, wood composition, and cellulose or lignin biosynthesis. Our study showed that during TW formation, genes involved in cellulose synthesis were induced, while the expression of lignin synthesis-related genes decreased, resulting in increased cellulose content and decreased lignin levels in TW. In addition, fasciclin-like arabinogalactan proteins play important role in TW formation. These findings may provide important insights into wood formation at the molecular level.

## Introduction

Trees are subjected to various constraints during development, including environmental constraints such as soil instability and damage caused by the actions of wind or snow, as well as biological constraints (such as limited light), which induce gravitropic and phototropic responses. To remain upright under adverse conditions, angiosperms have developed specialized wood tissue called tension wood (TW), which is formed in response to gravity. TW is formed on the upper sides of branches and leaning trunks of angiosperms and exerts a contractile force that allows a tree trunk to remain vertical or a branch to remain horizontal [1]. The other type of reaction wood is called compression wood (CW), which is formed on the ground-facing sides of trunks and the leaning stems of gymnosperns [2]. There are two major methods for artificially inducing the formation of tension wood, including bending the stem into a loop (extreme induction) and bending a stem using an immobile support. Both TW and opposite wood (OW) are generated with gravitropic stimuli, mechanical stimuli or both [2]. Compared with OW or normal wood (NW), TW exhibits some unusual characteristics [3]. Secondary cell walls of TW contain a gelatinous layer (G-layer), which consists of crystalline cellulose at a low microfibrillar angle [4]. The presence of a G-layer in TW results in the higher cellulose content and lower proportion of lignin found in TW compared with OW or NW. In addition, the G-layer also has a reduced proportion of vessel elements, and it is often formed more quickly than NW [5]. The formation of OW is either strongly retarded or completely inhibited.

To date, few studies have addressed the chemical composition of OW [6]. Some studies have demonstrated that OW has the same chemical composition as NW [7]. A comparison of gene expression during the formation of TW and OW would help to elucidate the process of wood formation. Andersson-Gunnerås *et al*. generated an expressed sequence tag library from TW-forming tissues in *Populus tremula* (L.) tremuloides (Michx.) and studied gene expression profiles during TW formation in *Populus tremula* (L.) in two growing seasons using microarray and metabolite analyses [8]. Jin *et al*. constructed a cDNA library from secondary xylem in the stem of a 2-year-old yellow poplar that was subjected to bending for 6 h at a 45° angle [9]. In total, 5,982 high-quality expressed sequence tags (ESTs) were generated, which were clustered into 1,733 unigenes, including 822 contigs and 911 singletons. Analysis of these ESTs identified many genes that are involved in cell wall biosynthesis and modification. Qiu *et al*. studied gene expression in *Eucalyptus nitens* branches bent at a 45° angle using microarrays that contained 4,900 cDNAs from xylem, and important genes involved in responses to gravitational stress in eucalyptus xylem were identified [10]. To characterize gene expression during TW formation in eucalyptus, cDNA array technology was employed. In total, 196 genes were found to be differentially regulated between control and bent wood samples. Some of these genes displayed distinctive expression patterns related to changes in secondary cell wall structure and composition. Analysis of these gene expression profiles provided new insights into the regulatory network of genes that are preferentially expressed in xylem [11]. Although these studies identified genes that are differentially expressed in TW versus controls, only a limited number of genes were investigated. However, little is known about the expression of genes associated with cell wall biosynthesis and modification on a large scale.

Next-generation (NG) sequencing techniques are low cost, high throughput sequencing methods that can generate information about numerous expressed genes in a short period of time. Platforms for NG sequencing include the Genome Analyzer (Solexa/Illumina), 454 (Roche) and ABI-SOLiD (Applied Biosystems) [12]. RNA-Seq is an effective approach to transcriptome profiling that uses NG sequencing technologies. RNA-Seq provides a far more precise measurement of the transcript levels of genes and their isoforms than other methods of analysis on a genomic scale [13]. Moreover, RNA-Seq can provide absolute gene expression measurements rather than relative measurements, thus overcoming many limitations of microarray analysis [14], [15].

*Betula platyphylla* Suk (birch) is a fast-growing woody species that it is tolerant to light, drought and flooding and adapts well to numerous types of soil. This widely grown tree is important in the biofuels and pulp industries. However, few essential regulatory genes have been identified to date. Such genes could be used for the genetic improvement of birch in particular to enhance its value as a source of biofuels and biomaterials. The aim of the present study was to investigate the molecular responses of birch to tension stimulus and lay the foundations for improving the wood properties of birch planted for the production of biofuels and pulp. The cellulose and lignin contents and wood anatomy of TW, OW and NW were analyzed in birch stems subjected to 8 weeks of mechanical bending. The results suggest that tension stimulus can influence xylem development in birch. As genes that are expressed early in the formation of reaction wood may significantly influence the subsequent quality of the wood, we examined the transcriptomes of TW, OW and NW in *B. platyphylla* subjected to bending for 2 weeks. We determined a complete set of birch xylem genes that were differentially expressed in response to gravitational stress and mechanical bending, and genes were identified that are involved in the biosynthesis of wood components, such as cellulose and lignin. The results of our study are potentially useful for improving wood properties in plants via genetic engineering.

## Materials and Methods

### Ethics Statement

The location for growing the birch trees used in this study is not required specific permission, because it is the experimental field of our Laboratory (State Key Laboratory of Tree Genetics and Breeding, Northeast Forestry University), which is named as “orchard of birch intensive breeding”, located in Northeast Forestry University. In this orchard, we have the experimental field, and all the birch trees used in this study were planted by us; therefore, we have the right to use these birch trees in study.

### Plant Material

Beginning in May 2011, 2-year-old birch trees were grown under natural conditions in a field in Harbin (the People’s Republic of China), located at 45°44′N and 126°36′E. All of the trees were well watered. TW formation was induced in trees during the most active period of growth by bending the tree and tying it to its neighbor tree in such a way that the midpoint of the stem was at an angle of approximately 45°; upright trees were used as the control. Two weeks after applying the stimulus, xylem samples were collected from two bent trees and two control trees. Tissues were collected by peeling the bark and scraping the exposed differentiating xylem (2–3 mm in depth) from both the upper (TW) and lower (OW) sides of the bent stems with a scalpel, as well as from corresponding locations on the controls (nonbent trees, NW). Xylem samples were immediately frozen in liquid nitrogen and stored at −80°C.

### Chemical Content and Wood Anatomy Analysis

Wood samples treated as described above were also harvested after 8 wks of bending to produce material for transverse sections (10–15 µm thick) and to analyze the cellulose and lignin contents. To characterize the presence of TW by the appearance of G-layers, sections were subjected to environmental scanning electron microscopy (ESEM). The cellulose and lignin contents were determined with a Fibertec™ 2010 & M6 system (Foss, Sweden) following the manufacturer’s protocol (www.foss.dk). Analyses were performed in triplicate. Analysis of variance (ANOVA) was performed using SPSS software.

### Construction of Illumina Library and Solexa Sequencing

Total RNA was isolated from OW, TW and NW using the CTAB method and digested with DNase I (RNase free) to remove DNA contamination. Since each treatment sample contains two trees, RNA was respectively isolated from these two trees and then pooled for library construction. Illumina Solexa sequencing using the GAII platform was carried out at the Beijing Genomics Institute (BGI; Shenzhen, China). Briefly, mRNA was isolated from total RNA from each sample using poly-T oligo-attached magnetic beads (Illumina) and sheared into small fragments using divalent cations (Illumina) at 94°C for 5 min. Complementary DNA (cDNA) was synthesized using mRNA fragments as templates and random primers as reverse complementary primers. To create blunt ends, the synthesized cDNAs were digested with T_4_ DNA polymerase and Klenow DNA polymerase. The 3′ ends of the blunt phosphorylated cDNA fragments were added with an adenosine (A) base using the polymerase activity of the Klenow fragment. Then, specific adaptors equipped with single ‘T’ base overhangs at their 3′ ends were ligated to the cDNA fragments supplemented with the ‘A’ base. The ligation products were run on a gel to select fragments of approximately 200 bp in length. These selected fragments were PCR amplified using a primer set that specifically annealed to the ends of the adapters. The PCR products were then purified with a QIAquick PCR Purification Kit that was used for construction of the Illumina library. After evaluation of the size, purity and concentration of the constructed library, single-stranded cDNA fragments were annealed to the flow cell surface in a cluster station (Illumina, San Diego, CA). Sequencing-by-synthesis was performed with an Illumina Genome Analyser, and the reads that passed default quality filtering parameters in the Illumina GA Pipeline GERALD stage were retained for further analysis.

### Data Analysis

High quality reads were generated by removing adaptor sequences, reads with only adaptor sequences, reads with unknown sequences ‘N’ and reads with a copy number of one (probably sequencing error). Transcriptome *de novo* assembly was performed in each library using the short oligonucleotide analysis program SOAPdenovo [16]. SOAPdenovo combines short reads with a certain length of overlap to form contigs, and the reads are mapped back to the contigs. From the paired-end reads, contigs from the same transcript and the distances between these contigs were both detected. Scaffolds were formed by connecting the contigs from the same transcript using SOAPdenovo, with N representing unknown sequences between the two contigs. Paired-end reads were employed for gap filling within scaffolds to generate sequences with the fewest Ns that could not be extended at either end. Such sequences were considered to be tentative unigenes (TUGs). The TUGs from each library were further assembled from a single set of nonredundant unigenes using TGICL software [17]. The abundance of each gene was determined by calculating the reads per kb per million reads (RPKM) value, as described by Mortazavi *et al*. [18].

### Functional Annotation

Nonredundant unigenes were subjected to BLASTX analysis against the NR of NCBI and Swiss-Prot protein databases to search for similarity. The unigenes with BLASTX E-value >10^−5^ were discarded during functional annotation. Gene Ontology (GO) annotation was assigned using the Blast2go program [19]. After GO annotation was assigned for each unigene, WEGO software was used to perform GO functional classification for all unigenes according to molecular function, biological process and cellular component [20].

### Real-time Quantitative RT-PCR Analysis

To test the overall validity of the expression levels of genes determined by DGE analysis, real time RT-PCR was performed. One microgram of total RNA from each sample was reverse transcribed using oligo-deoxythymidine as a primer in a 10 µl volume. Real time RT-PCR was performed using an MJ Opticon™^−2^ machine (Bio-Rad, Hercules, CA). *Actin* (GenBank number: HO112155) and *ubiquitin* (GenBank number: HO112156) genes were used as internal controls. The reaction mixture (25 µl) contained 12.5 µl of SYBR Green Real-time PCR Master Mix (Toyobo), 0.5 µM of forward or reverse primers (Table 1 in File S1), and cDNA template (equivalent to 100 ng of total RNA). The amplification procedure was performed using the following parameters: 94°C for 30 s; 45 cycles at 94°C for 12 s, 58°C for 30 s, 72°C for 45 s and 80°C for 1 s for plate reading. A melting curve was generated for each sample to assess the purity of the amplified products. All experiments were carried out with three biological replicates. The expression levels were calculated from the threshold cycle using the delta-delta CT method.

## Results and Discussion

### Cellulose and Lignin Content and Wood Anatomy

**Figure 1:**
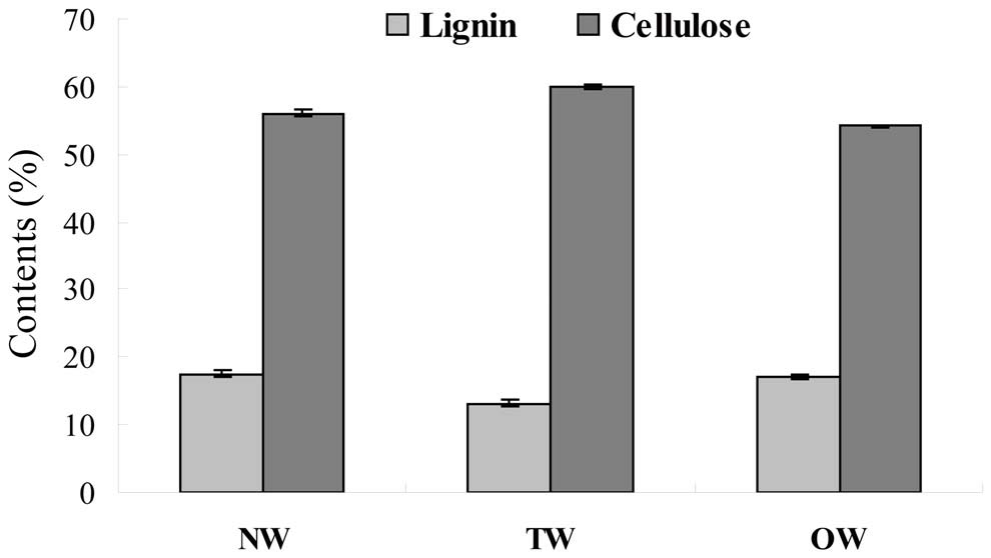
Cellulose and lignin contents of TW, OW and NW. * Significant differences (P<0.05) between TW and OW or NW. Cellulose and lignin content are expressed as a percentage of cell wall residue (CWR).

The cellulose and lignin contents of TW, OW and NW were analyzed in the stems of 2-year-old birch trees subjected to mechanical bending for 2 and 8 weeks. There was no significant difference in the cellulose and lignin contents among the three tissues subjected to bending for 2 weeks (data not shown). However, after bending for 8 weeks, the cellulose content was significantly higher in TW than in OW or NW, whereas the lignin content was significantly lower in TW than in the other types of wood (Figure 1).

**Figure 2:**
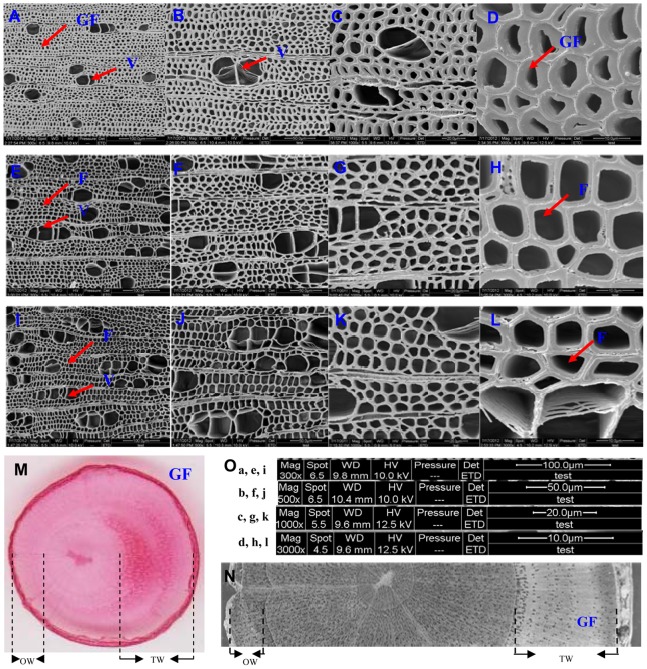
Images of wood anatomy of TW, OW and NW. Environmental scanning electron microphotographs of the transverse surface of tension wood (A–D) and opposite wood (E–H) in stems (LS) of birch cultivated for 8 wks under control conditions (I–L). A, E, I: ×300; B, F, J: ×500; C, G, K: ×1000; D, H, L: ×3000. Scale bars are described in O. V, vessel; F, fibre; GF, G-layer fibre. M and N show the unusual growth of different parts of birch stems subjected to bending for 8 wks.

Samples were collected from the upper (TW) and lower (OW) sides of bent 2-year-old trees and from straight trees (NW) after 8 weeks of bending. Sections were produced from these samples and examined. There were obvious differences in growth rates between TW and OW, which led to the unusual growth of birch stems (Figure 2M, N). In addition, TW exhibited characteristic fibers with an additional G-layer (Figure 2A–D) and contained fewer vessels with smaller lumens than OW (Figure 2E–H) or NW (Figure 2I–L). No G-layer was present in NW, or in OW subjected to bending for 8 weeks (Figure 2E–L). The increased cellulose content and fiber numbers in response to tension is thought to be part of a stem-sensing mechanism, which leads to increased tensile strength to counteract the gravitational forces acting on the leaning stem.

### *De novo* Transcriptome Assembly

The gene expression in response to artificial bending were usually studied after bending for 6 h to 3 weeks [8], [9], [11], and that period is important for study the tension wood formation. Three cDNA libraries were constructed from *B. platyphylla* TW, OW and NW, after bending for two weeks (referred to as the TW, OW and NW libraries). In total, 52,139,846, 54,878,244 and 54,696,478 clean reads were generated from the TW, OW and NW libraries, respectively. The data of clean reads can be accessed at the NCBI Sequence Read Archive under the accession number SRA053683 (SRR513429, SRR520271, SRR520272). The reads from each library were assembled into contigs using Trinity software [21], and the contigs were further assembled into tentative unigenes (TUGs). In total, 77,783, 66,909 and 74,068 TUGs were generated from the TW, OW and NW libraries, respectively (Table 2 in File S1). Finally, all unigenes from the three libraries were assembled together to form a single set of non-redundant unigenes using TGICL software [17]. A total of 80,909 non-redundant unigenes (GenBank No.: KA198535-KA279443) was generated, with a mean size of 768 nt and a total length of 62,112,335 nt. The distribution of unigene lengths is shown in Table 1.

**Table 1:** Length distribution of all unigenes.

Length of non-redundant unigenes	Total Number	Percentage
100–500 nt	45,950	56.79%
500–1000 nt	15,524	19.19%
1000–1500 nt	7,945	9.82%
1500–2000 nt	4,940	6.11%
> = 2000 nt	6,550	8.09%
N50 [1]	1309	
Mean	768	
All Unigene	80,909	
Length of all Unigene (nt)	62,112,335	

*N50 = median length of all unigenes.

*Mean = average length of all unigenes.

### Annotation of Nonredundant Unigenes

Among the non-redundant genes, 43,245 and 29,977 genes showed significant similarity to known proteins in the Nr and Swiss-Prot databases, respectively.

GO classification was performed to study the functional group distribution of non-redundant unigenes (Table 3 in File S1 & Figure S1). In all three libraries, cellular components were the most abundant category, followed by biological processes and molecular function. Of the three principal gene ontologies, the most abundant subcategories in the three libraries were ‘all cell’, ‘cell part’, ‘catalytic activity’, ‘binding’, ‘organelle’, ‘metabolic process’ and ‘cellular process’, suggesting that these functional processes play major roles in TW, OW and NW.

### The Most Abundant Genes

Genes that are abundant in certain tissues or development stages may be important for the tissue or developmental stage; therefore, we identified the ten most abundant genes in the TW, OW and NW libraries (Table 2). Seven transcripts were highly abundant in all of the libraries, including two lipid transfer proteins (LTP), allergenic isoflavone reductase-like, ag13, polyubiquitin, cytochrome P450 like_TBP and hypothetical protein (Table 2). These findings suggest that these genes play essential roles in wood formation. Genes encoding LTP, cytochrome P450 like_TBP, rRNA intron-encoded homing endonuclease, phi-1 and C3HL domain class transcription factor were abundant in OW, with much less abundance in TW, suggesting that these genes play important roles in OW development rather than TW development. On the contrary, genes encoding FLA, glycine-proline rich protein and class II chitinase were highly abundant in TW, with little expression in OW; obviously, these genes play important roles in TW formation.

**Table 2:** Expression profiles of the top 10 abundant genes in TW, OW and NW.

GenBank Number:	RPKM			Functional annotation
	TW	OW	NW	
NW				
KA275752	3724.332	15053.39	13608.72	lipid transfer protein family protein
KA250254	7559.774	9818.979	11268.71	allergenic isoflavone reductase-like
KA244244	6013.817	11912.6	9884.284	lipid transfer protein (LTP) family protein
KA200383	8000.385	6803.264	6643.729	ag13
KA262474	2025.879	2844.252	5724.919	xylem sap protein 10 kDa
KA201101	5649.985	4611.468	5311.726	polyubiquitin
KA259905	3619.965	13164.14	5246.218	cytochrome P450 like_TBP
KA250879	4966.727	5211.61	4390.03	hypothetical protein
KA257282	6723.616	2745.166	3908.591	glycine-proline rich protein
KA257879	2550.106	2733.757	3309.829	polyubiquitin containing 7 ubiquitin monomers
TW				
KA200383	8000.385	6803.264	6643.729	ag13
KA250254	7559.774	9818.979	11268.71	allergenic isoflavone reductase-like protein
KA219691	7317.225	242.5356	654.6115	fasciclin-like arabinogalactan protein
KA257282	6723.616	2745.166	3908.591	glycine-proline rich protein
KA244244	6013.817	11912.6	9884.284	lipid transfer protein (LTP) family protein
KA201101	5649.985	4611.468	5311.726	polyubiquitin
KA250631	5167.007	2003.051	2410.727	class II chitinase
KA250879	4966.727	5211.61	4390.03	hypothetical protein
KA275752	3724.332	15053.39	13608.72	lipid transfer protein family protein
KA259905	3619.965	13164.14	5246.218	cytochrome P450 like_TBP
OW				
KA275752	3724.332	15053.39	13608.72	lipid transfer protein family protein
KA259905	3619.965	13164.14	5246.218	cytochrome P450 like_TBP
KA244244	6013.817	11912.6	9884.284	lipid transfer protein (LTP) family protein
KA250254	7559.774	9818.979	11268.71	allergenic isoflavone reductase-like protein
KA259904	2660.703	7896.881	3299.789	rRNA intron-encoded homing endonuclease
KA200383	8000.385	6803.264	6643.729	ag13
KA250879	4966.727	5211.61	4390.03	hypothetical protein
KA201101	5649.985	4611.468	5311.726	polyubiquitin
KA249327	954.0103	2977.693	845.0917	phi-1
KA262532	779.1426	1631.054	1012.204	C3HL domain class transcription factor

### Identifying Differentially Expressed Genes in TW, OW and NW

**Figure 3:**
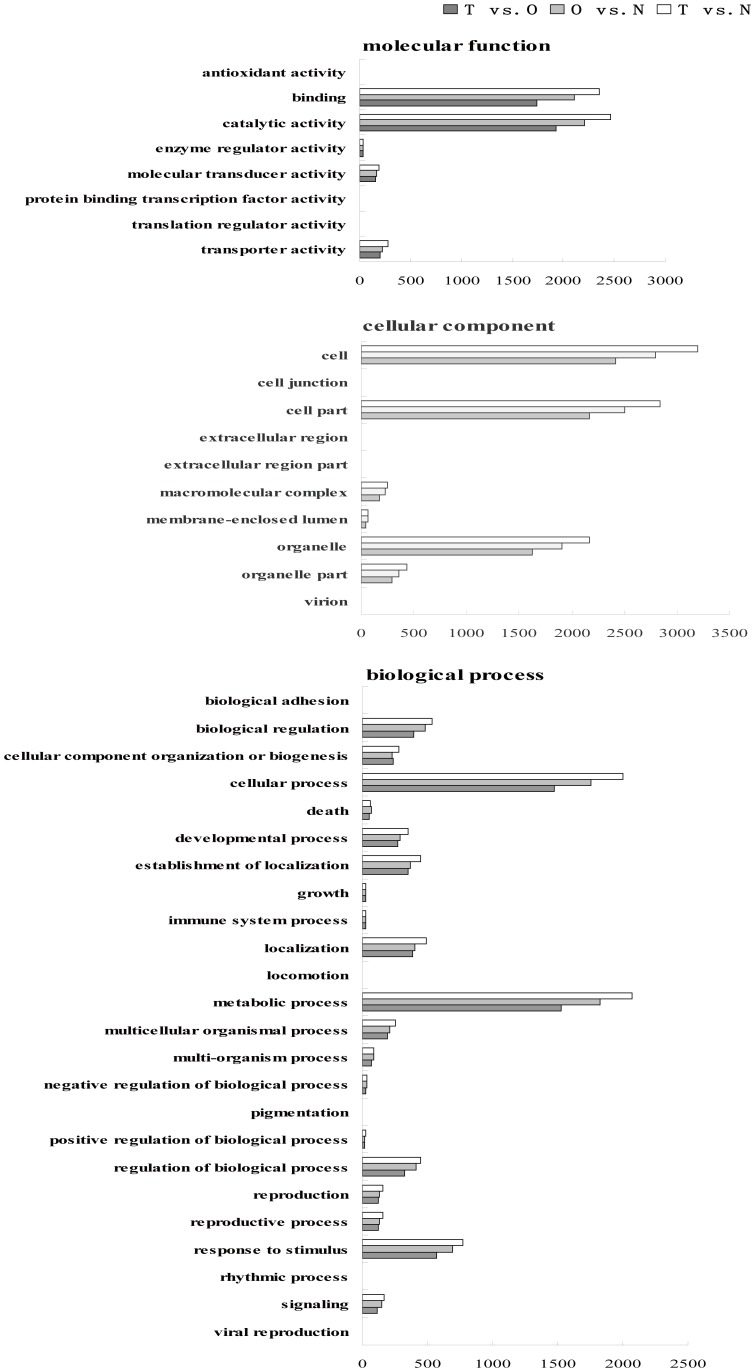
Gene ontology (GO) categories assigned to differentially expressed genes in response to artificial bending. The x-axis shows the number of unigenes, and the y-axis shows the GO subcategories.

Unigenes more than 400 bp in length, FDR ≤0.0001 and the absolute value of |log2Ratio| ≥2, were identified as significantly differentially expressed using DGE analysis (Table S1). The distributions of differentially expressed genes among the three libraries are shown in Table 3. GO classification was performed to study the functional group distribution of the differentially expressed genes (Figure 3). Among the three principal gene ontologies, the most abundant subcategories in TW, NW and OW were ‘all cell’, ‘cell part’, ‘catalytic activity’, ‘binding’, ‘organelle’, ‘metabolic process’ and ‘cellular process’, thereby suggesting that these functional processes play major roles in the formation of TW and OW.

**Table 3:** The distributions of differentially expressed genes.

	Number of genes		
	TW vs.NW	OW vs.NW	OW vs.TW
Total	6264	4533	4025
Up-regulated	2792	2322	2470
Expressed only in this tissue	525	313	323
Down-regulated	3472	2211	1555
Expressed only in control tissue	501	315	480

*The control tissue refers to the latter tissue in every column.

### Fasciclin-like Arabinogalactan Proteins are Abundant and Upregulated in TW

**Figure 4:**
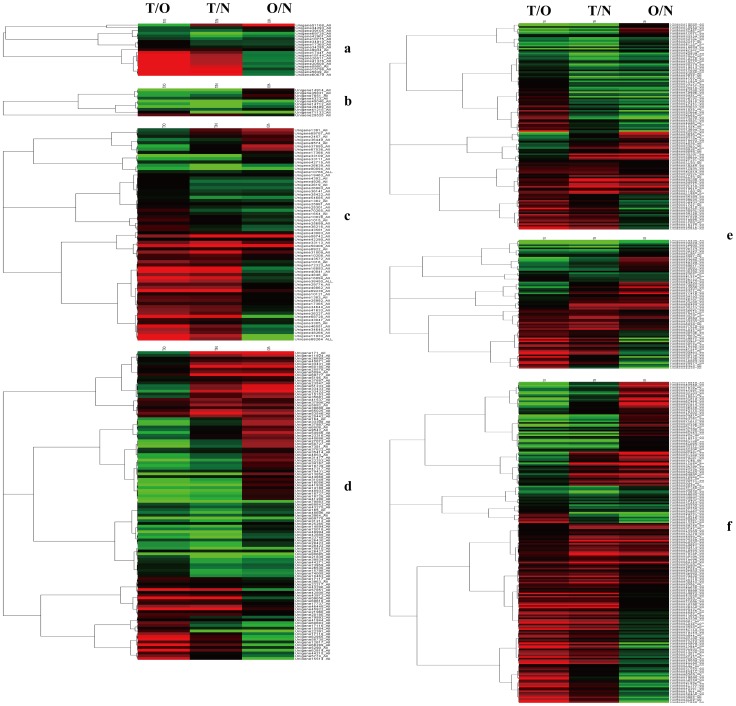
Hierarchical cluster analysis of different gene families with altered expression levels in response to artificial bending. All ratios are log2 transformed so that inductions and repressions of identical magnitudes are numerically equal but opposite in direction. Log ratios of 0 (ratios of 1) are shown in black, and increasingly positive (induction) or negative (repression) log ratios are shown in red or green, respectively, with increasing intensities. Red indicates induction, and green indicates repression in arrays. A: FLA family genes, B: LTP family genes, C: Cellulose biosynthesis related genes, D: Lignin biosynthesis related genes, E: TFs, F: Growth hormone-related genes.

Our results show that a fasciclin-like arabinogalactan protein (FLA) gene (KA219691) is one of the top 10 most abundant genes in the TW library and is strongly induced in TW relative to OW and NW (Table 2). In addition, 19 unique FLAs genes were identified, and 10 FLAs were induced in TW compared with NW and OW (Table 4 in File S1 & Figure 4A), suggesting that these genes are involved in TW formation. *FLA*s are a subclass of AGPs containing domain(s) that are involved in wood formation [22] and the building of the G-layer [23]. Taken together, these results suggest that these *FLAs* are involved in building the G-layer in TW of birch.

### LTP may Play Specific Roles in TW Formation

LTPs (lipid transfer proteins) are involved in promoting plant cell wall loosening and thus may play a role in cell expansion and plant growth [24]. We identified 10 unique LTP genes, and 2 LTP genes (KA275752 and KA244244) were among the top 10 most abundant genes in all three libraries (Table 2), suggesting that they play crucial roles in the formation of TW, OW and NW. In addition, 7 *LTP* genes had lower levels of expression in TW than in OW or NW (Table 4 in File S1& Figure 4B). Combined with the smaller lumen in TW cells, the decreased expression of these LTPs in TW may contribute to decreased cell wall loosening or inhibited cell expansion during TW formation.

### Genes Involved in Cellulose Synthesis Were Induced during TW Formation

Cellulose, which comprises long chains of β-1, 4-linked glucosyl residues, is formed from uridine diphospho (UDP)–glucose catalyzed by cellulose synthase (CesA, EC2.1.4.12) [25]. Our results show that 12 CesA genes were highly expressed in TW relative to in OW or NW (Table 4 in File S1, Figure 4C & Figure S2a). The unigenes KA240937, KA240936 and KA207874 shared high identities with the irregular xylem3 (irx3) from Arabidopsis, which was required for secondary cell wall synthesis [26], and all of the three genes were upregulated in TW compared those in OW or NW (Table 4 in File S1 & Figure 4C). Moreover, our results show that cellulose levels in TW are elevated compared with in OW and NW (Figure 1). Taken together, these results suggest that the increased expression level of *CesA* results in elevated cellulose levels; therefore, these *CesA* genes play roles in the synthesis of cellulose and are involved in TW formation.

Cellulose biosynthesis rosettes use UDP-glucose as a substrate [27], and the UDP-glucose synthesis pathways involve a serial enzymes, such as sucrose synthase (SuSy), sucrose phosphate synthase (SPS), UDP-glucose 4-epimerase (GALE), glucose-1-phosphate uridylyltransferase and a-expansins [28]–[32]. Our results show that these genes were upregulated in TW than in NW or OW (Table 4 in File S1, Figure 4C & Figure S2a). For instance, one *SuSy* genes was significantly induced, and three *SuSy* were slightly enriched in TW compare with OW or NW (Table 4 in File S1 & Figure 4C). Six *SPSs* were identified, and 5 of them were highly induced in TW compared with OW or NW. Two *GALE* genes were highly enriched in TW. Two *glucose-1-phosphate uridylyltransferase* genes were significantly enriched in TW compared with in OW or NW. Moreover, 3 *a-expansin* genes (KA276137, KA202593 and KA270930) were highly induced in TW relative to in OW (Table 4 in File S1 & Figure 4C). The fact that many cellulose biosynthesis-related genes were induced in TW, combined with the high levels of cellulose that accumulated in TW, clearly suggest that cellulose biosynthesis is highly activated in TW. Therefore, the maintenance of high levels of cellulose biosynthesis is important for TW formation.

### Genes involved in the Shikimate, Phenylpropanoid and Monolignol Biosynthesis Pathways were Downregulated in TW

The lignin content decreased significantly in TW subjected to bending for 8 weeks compared with NW and OW (Figure 1), which indicates that the lignin biosynthesis pathway is inhibited in TW during the bending stress period. The shikimate pathway functions in lignin biosynthesis by channeling the flow of carbon from sugar metabolism to the biosynthesis of phenylalanine. Phenylalanine, the precursor of monolignol, is converted into monolignol *via* the phenylpropanoid and monolignol biosynthesis pathways [33], [34]. The shikimate pathway includes the enzymes dehydroquinate dehydratase-shikimate dehydrogenase (DHQ-SDH), shikimate kinase (SK), 5-enolpyruvylshikimate-3-phosphate synthase (EPSPS) and others. Most of genes involved in the shikimate pathway were downregulated in TW-forming tissues relative to OW and NW (Table 4 in File S1, Figure 4D & Figure S2b). For instance, 7 *DHQ-SDH* genes were downregulated, while only 2 *DHQ-SDH* genes were upregulated in TW relative to OW or NW. Two SK genes were significantly downregulated in TW compared with in OW or NW. Two *EPSPS* genes were downregulated in TW compared with OW or NW. In the TW of birch, the expression of *DHQ-SDH*, *SK* and *EPSP* were all downregulated, and lignin content in TW was also decreased (Table 4 in File S1, Figure 4D & Figure 1). Taken together, these results suggested that the shikimate pathway is positively regulated during lignin biosynthesis in both angiosperm and gymnosperm plants.

The biosynthesis of monolignols proceeds through the phenylpropanoid pathway, which starting with the domination of phenylalanine to produce cinnamic acid, which then forms monolignols *via* the monolignol biosynthetic pathway. The genes involved in the early phenylpropanoid pathway were downregulated in TW compared with in OW and NW (Table 4 in File S1, Figure 4D & Figure S2b). For instance, PAL (phenylalanine ammonialyase) is the first enzyme in the phenylpropanoid pathway. and four *PAL* genes (KA266994, KA273229, KA277908 and KA243885) were expressed with lower expression levels in TW than in OW or NW. The enzyme 4CL (4-coumarate-coa ligase ) is the key enzyme in the phenylpropanoid pathway, and eight *4CLs* were downregulated in TW compared with in NW or OW. In addition three *CCoAOMT* (caffeoyl-CoA O-methyltransferase) and six *CAD* (cinnamyl alcohol dehydrogenase) genes were highly downregulated in TW relative to in NW or OW. The decreased transcript levels of genes involved in the phenylpropanoid and monolignols biosynthesis pathways combined with decrease of lignin content suggest that lignin biosynthesis is inhibited during TW formation, and these genes may play important roles during TW formation in birch.

POD (peroxidase) and laccase are involved in monolignol polymerization and modifications. The transcript levels of 20 *POD* genes were downregulated in TW compared with in NW or OW. The transcript levels of 4 laccase genes (KA265956, KA201153, KA207714 and KA211912) were significantly decreased in TW compared with in OW or NW. These results indicate that monolignol polymerization and modifications are inhibited during TW formation.

From the above results, it is evident that the genes involved in the shikimate, phenylpropanoid and monolignol biosynthesis pathways are generally downregulated in TW. In addition, the lignin content was decreased in the TW of birch. Taken together, these results suggest that the lignin biosynthesis is inhibited in TW by the downregulation of genes involved in the shikimate, phenylpropanoid and monolignol biosynthesis pathways.

### Identification of Wood-related Transcription Factors

We focused our study on NAC and MYB TFs, because they play important roles in secondary cell wall modification [35]–[38]. Using hierarchical cluster analysis, differentially expressed *NAC* genes were assigned into two groups according to their expression patterns (Table 4 in File S1 & Figure 4E): one group contained the *NACs* that were upregulated in TW and another included NACs that were downregulated in TW compared with OW or NW. The Unigenes KA246202 and KA269898 matched *VND1* from *Arabidopsis*, and both of them were upregulated in TW compared in OW or NW (Table 4 in File S1 & Figure 4E). The Unigene KA255359 matched *Arabidopsis NST1*, a key regulator of the formation of secondary walls [39], and its transcript level was altered significantly between OW, TW and NW, suggesting that it is involved in the formation of secondary walls. The changes in *NAC* expression among TW, OW and NW suggest that *NACs* are closely associated with TW formation, and they may play roles in mediating TW development. *MYB* family proteins play positive or opposite roles in the lignin biosynthetic process [40], [41]. Nearly half of the *MYBs* were upregulated in TW vs. OW or NW, and the remaining *MYBs* were downregulated in TW compared with OW and NW (Table 4 in File S1 & Figure 4E). Unigenes KA244417, KA275422 and KA275423 all matched *AtMYB4*, which is a key repressor of the phenylpropanoid pathway involved in the repression of lignin synthesis [42]. These three genes are upregulated in TW (Table 4 in File S1 & Figure 4E); meanwhile, lignin content were both decreased in TW (Figure 1), which suggested that they may have similar functions as *AtMYB4* in the repression of lignin synthesis. Unigene KA262304 matched *AtMYB46*, a key gene in the regulation of secondary wall biosynthesis in *Arabidopsis* [38]. As it is upregulated in TW, it may be involved in the formation of TW. The differences in *MYB* expression between TW, OW and NW suggests that these MYB proteins may play positive or opposite roles in the process of TW formation.

### The Expression of Growth Hormone-related Genes

Auxin is involved in acid-induced wall loosening and vascular development [43] and may be a key regulator of TW formation. A large number of auxin family genes were identified, including genes that encoded ARF (Auxin response factor), Aux/IAA protein, auxin efflux carrier protein, auxin influx transport protein, auxin response factor and auxin-induced protein (Table 4 in File S1). Most auxin family genes were differentially expressed among TW, NW and OW (Table 4 in File S1 & Figure 4F), and were upregulated in TW compared in OW and NW, which implied that auxin-related genes are involved in the development of birch TW.

### Confirmation of the DGE Results by qRT-PCR Analysis

To evaluate the validity of expression profiles detected by the transcriptome study, real-time RT-PCR analysis was performed. The real-time RT-PCR results were in agreement with the DGE results (Figure S3), which validated the reliability of the DGE results.

## Conclusions

In this study, our results showed that the cellulose content was significantly increased in TW compared with OW or NW, whereas the lignin content in TW was significantly decreased. Three transcriptome libraries were constructed from TW, OW and NW, and 9,684 significantly differentially expressed genes were identified. Among these, genes were identified that were involved in secondary cell wall structure and wood composition, including cellulose and lignin biosynthetic genes. The characterization of gene expression in response to artificial bending on a genomic level will accelerate our understanding of the mechanisms that underlie xylem development in plants and will help us to identify genes involved in xylem development in birch. These findings may have potential applications for the improvement of wood properties in plants via genetic engineering.

## Supporting Information

Figure S1 **Result of GO analysis based on biological process (a), cellular components (b) and molecular function (c) of the three birch transcriptomes.**
(TIF)

Figure S2 **Pathway analysis of cellulose and lignin biosynthesis.** Regulation of genes involved in cellulose (a) and lignin (b) synthesis during tension wood (TW) formation. The figure illustrates the sucrose, galactose, glucose and fructose metabolic pathways, which are related to cellulose synthesis and the shikimate, phenylpropanoid and monlignol biosynthetic pathways, which are related to lignin synthesis, according to http://www.genome.jp/kegg/pathway/map. Modifications of transcript abundance (Table 4 in File S1 and Fig. 4) are indicated by the following colors: red, increase; blue, decrease; gray, not present in the three libraries; black, not affected. Genes that were significant for wood formation were identified according to signal strength and their relative abundance in the libraries. The following genes were included: cellulose synthase (CesA); sucrose synthase (SuSy); sucrose phosphate synthase (SPS); sucrose-phosphatase and sucrose phosphate phosphatase (SPP); UDP-glucose 4-epimerase (GALE); glucose-1-phosphate uridylyltransferase; phosphoglucomutase; glucose-6-phosphate isomerase; hexokinase and fructokinase; beta-fructosidases; dehydroquinate dehydratase-shikimate dehydrogenase (DHQ-SDH), shikimate kinase (SK), 5-enolpyruvylshikimate-3-phosphate synthase (EPSPS); phenylalanine ammonialyase (PAL); 4-coumarate-coa ligase (4CL); cinnamyl alcohol dehydrogenase (COMT); caffeoyl-CoA O-methyltransferase (CCoAOMT); cinnamyl alcohol dehydrogenase (CAD); Cinnamoyl-CoA reductase (CCR) and peroxidase (POD).
(TIF)

Figure S3 **Confirmation of Solexa expression profiles by qRT-PCR analysis.** NW was the control. All ratios are log2 transformed.
(TIF)

Table S1 **Differentially expressed genes identified by digital gene expression (DGE) analysis (>400 bp, FDR ≤0.0001, |log2 Ratio| ≥2).**
(XLS)

File S1 **Contains Tables 1, 2, 3, and 4. Table 1.** Primer sequences used for real time RT-PCR. **Table 2.** General characteristics of the three transcriptomes. **Table 3.** Results of GO analysis based on biological process, cellular components and molecular functions of the three birch transcriptomes. **Table 4.** Hierarchical cluster analysis of gene families that were differentially expressed in response to artificial bending. All gene families described in the Discussion are listed, including genes that were differentially expressed and those that were not. The differentially expressed genes were identified by a |log2Ratio| ≥1.
(DOC)
