# Supplementary figures and images for: Comprehensive Transcriptome Analysis of Developing Xylem Responding to Artificial Bending and Gravitational Stimuli in Betula platyphylla
Source: PLoS One. 2014 Feb 20;9(2):e87566. doi: 10.1371/journal.pone.0087566 (PMC3930542; doi:10.1371/journal.pone.0087566)

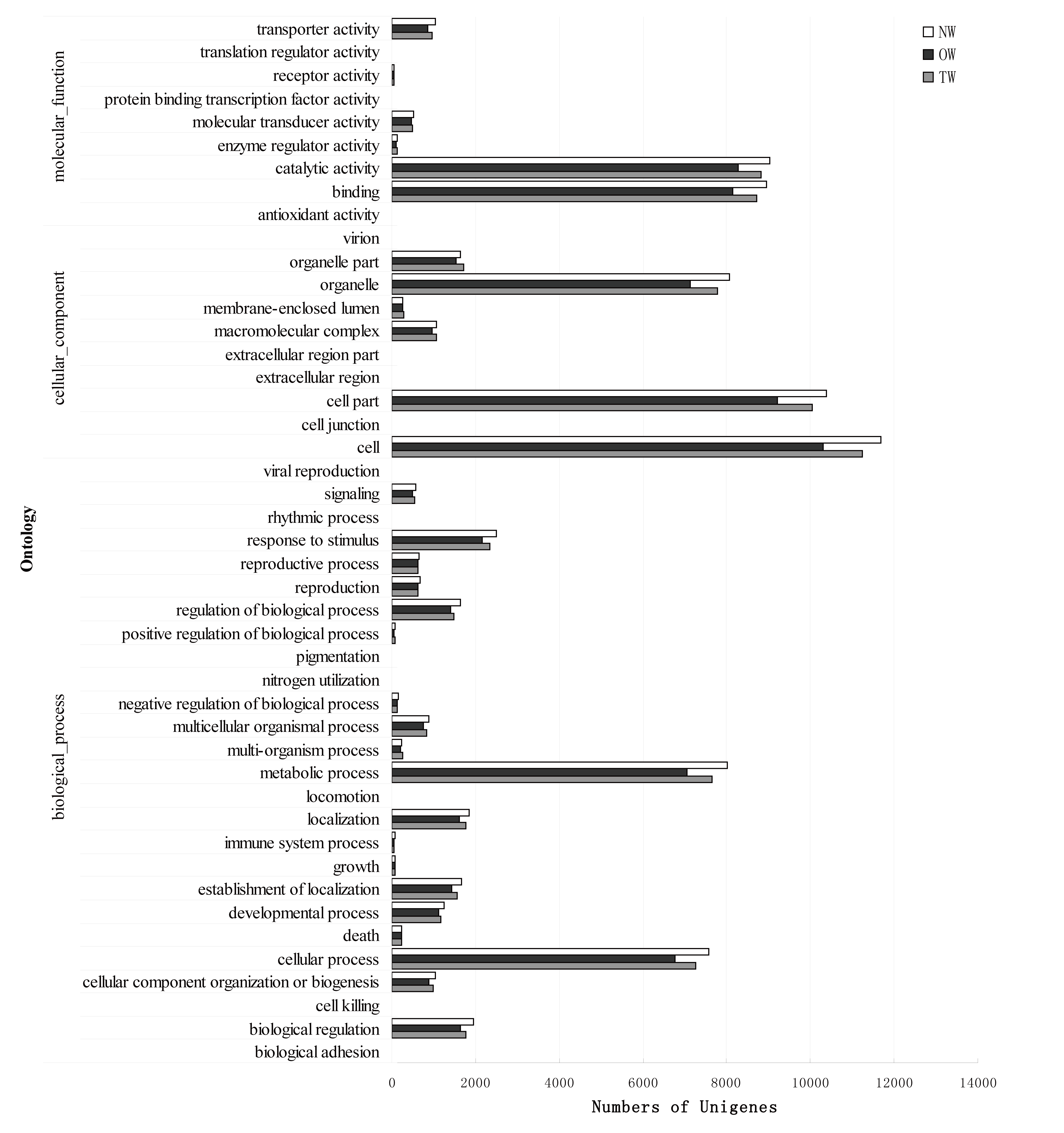

Supplement: Figure S1 — Result of GO analysis based on biological process (a), cellular components (b) and molecular function (c) of the three birch transcriptomes. (TIF) [file pone.0087566.s001.tif]

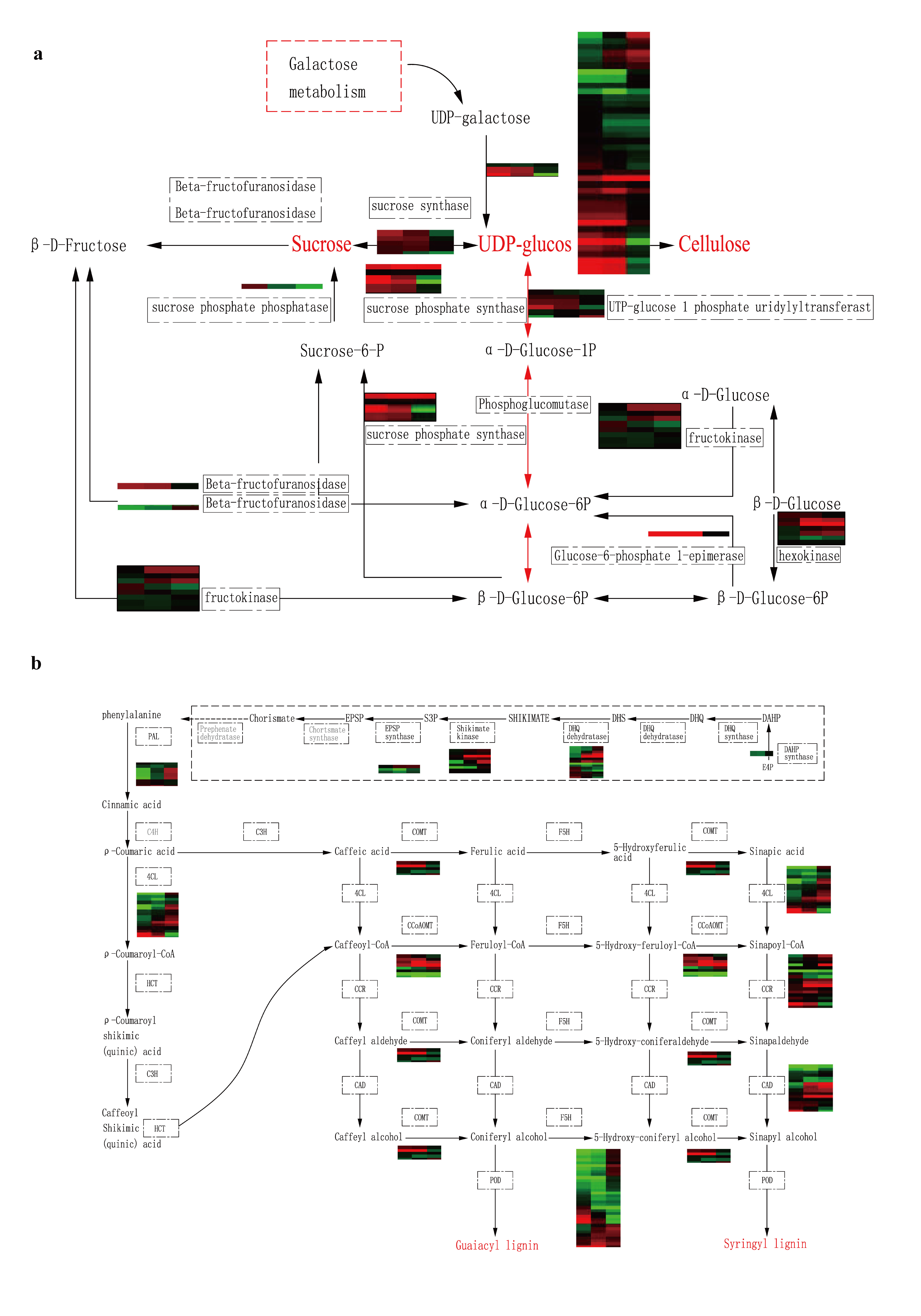

Supplement: Figure S2 — Pathway analysis of cellulose and lignin biosynthesis. Regulation of genes involved in cellulose (a) and lignin (b) synthesis during tension wood (TW) formation. The figure illustrates the sucrose, galactose, glucose and fructose metabolic pathways, which are related to cellulose synthesis and the shikimate, phenylpropanoid and monlignol biosynthetic pathways, which are related to lignin synthesis, according to http://www.genome.jp/kegg/pathway/map. Modifications of transcript abundance (Table 4 in File S1 and Fig. 4) are indicated by the following colors: red, increase; blue, decrease; gray, not present in the three libraries; black, not affected. Genes that were significant for wood formation were identified according to signal strength and their relative abundance in the libraries. The following genes were included: cellulose synthase (CesA); sucrose synthase (SuSy); sucrose phosphate synthase (SPS); sucrose-phosphatase and sucrose phosphate phosphatase (SPP); UDP-glucose 4-epimerase (GALE); glucose-1-phosphate uridylyltransferase; phosphoglucomutase; glucose-6-phosphate isomerase; hexokinase and fructokinase; beta-fructosidases; dehydroquinate dehydratase-shikimate dehydrogenase (DHQ-SDH), shikimate kinase (SK), 5-enolpyruvylshikimate-3-phosphate synthase (EPSPS); phenylalanine ammonialyase (PAL); 4-coumarate-coa ligase (4CL); cinnamyl alcohol dehydrogenase (COMT); caffeoyl-CoA O-methyltransferase (CCoAOMT); cinnamyl alcohol dehydrogenase (CAD); Cinnamoyl-CoA reductase (CCR) and peroxidase (POD). (TIF) [file pone.0087566.s002.tif]

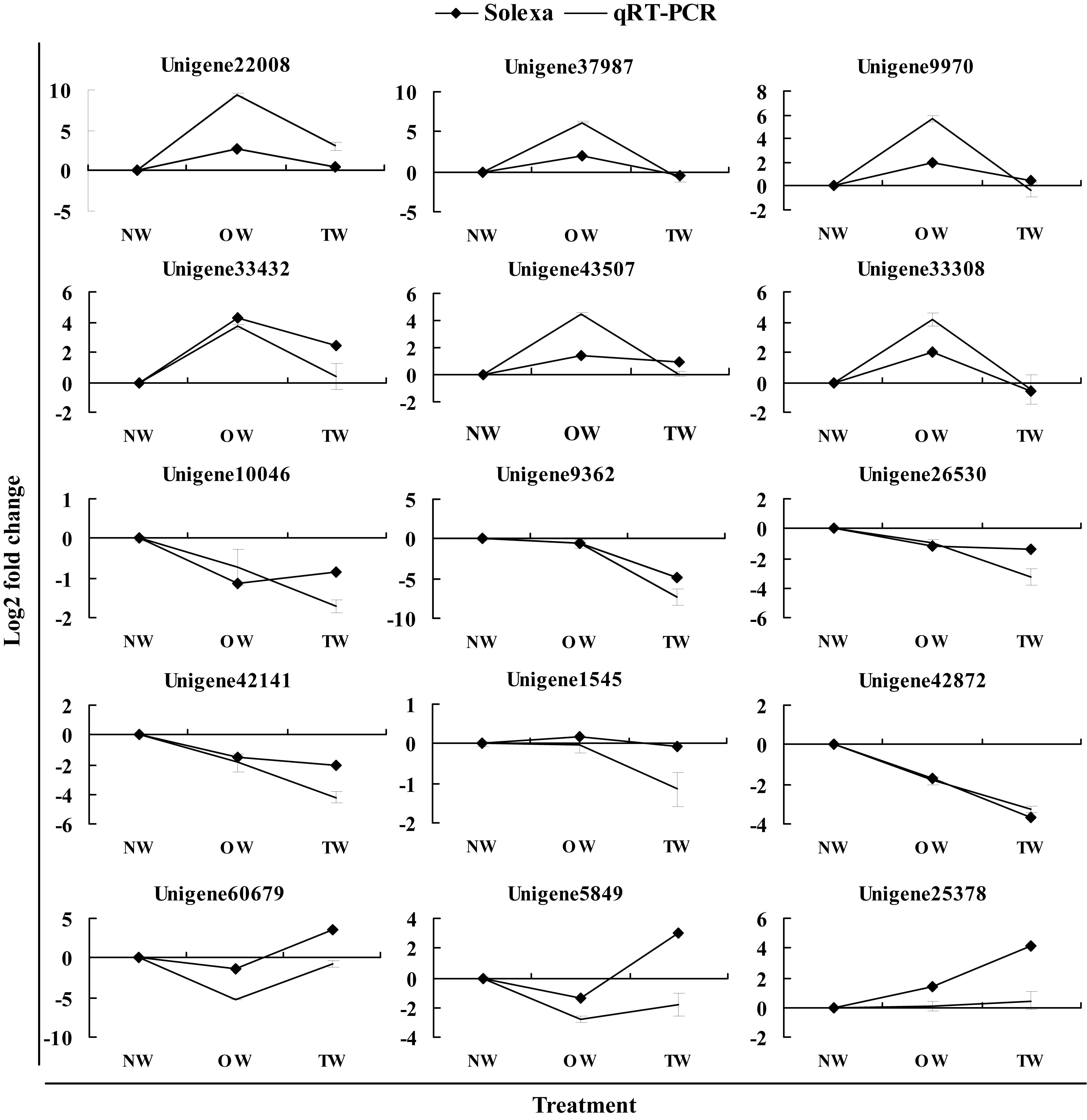

Supplement: Figure S3 — Confirmation of Solexa expression profiles by qRT-PCR analysis. NW was the control. All ratios are log2 transformed. (TIF) [file pone.0087566.s003.tif]
