# Supplementary material for: Comprehensive Transcriptome Analysis of Developing Xylem Responding to Artificial Bending and Gravitational Stimuli in Betula platyphylla
Source: PLoS One. 2014 Feb 20;9(2):e87566. doi: 10.1371/journal.pone.0087566 (PMC3930542; doi:10.1371/journal.pone.0087566)
Supplement: File S1 — Contains Tables 1, 2, 3, and 4. Table 1. Primer sequences used for real time RT-PCR. Table 2. General characteristics of the three transcriptomes. Table 3. Results of GO analysis based on biological process, cellular components and molecular functions of the three birch transcriptomes. Table 4. Hierarchical cluster analysis of gene families that were differentially expressed in response to artificial bending. All gene families described in the Discussion are listed, including genes that were differentially expressed and those that were not. The differentially expressed genes were identified by a |log2Ratio| ≥1. (DOC) [file pone.0087566.s005.doc]

**Table 1 Primer sequences used for real time RT-PCR assays.**

| GenBank number: | Gene ID | Primer sequence (5’–3’) |
| --- | --- | --- |
| KA261929 | >Unigene22008_All | 5’-GCCAGGTCTCACCTCTTCAG  3’-AGCAAGGTTCTTTTCGTCCA |
| KA277908 | >Unigene37987_All | 5’-GGGAAAGATTCGAGAAGGGA  3’-AAAGACTCCTCCCCCATTTG |
| KA249891 | >Unigene9970_All | 5’-TTTCACGTTCGGGTGGTTTC  3’-GTTGAATCTTCCCGGTAGCA |
| KA273353 | >Unigene33432_All | 5’-TGGATGCGGATGGTACTGTT  3’-TGTCTGCACCAAGCAAACTC |
| KA202519 | >Unigene43507_All | 5’-GGACGACGCAAAATAGAATG  3’-AACTTCGTCCAAGCATGACA |
| KA273229 | >Unigene33308_All | 5’-CAGTACCTGGCCAATCCTGT  3’-TTTTCAGCCTTGGGATGATC |
| KA249967 | >Unigene10046_All | 5’-GACGCAGTCCTTTTGTGTCA  3’-CTACAAAACGCCGTTGGATT |
| KA249283 | >Unigene9362_Al | 5’-CCGATCGTTCAACGGGAAAG  3’-CAACACCATCGTCTTCCTCTG |
| KA266451 | >Unigene26530_All | 5’-GCCTGAACACCACCGATCTA  3’-AAGCAGTCTGGTCAGCACTG |
| KA201153 | >Unigene42141_All | 5‘-ATTGCCTCAACACCTTTTGC  3‘-GGCAGGCGTCTGTAATAAGC |
| KA241466 | >Unigene1545_All | 5’-ATGCCAGTGCCCCAGGTGCA  3’-GGCATCGCCTGGTTTTATTA |
| KA201884 | >Unigene42872_Al | 5’-GGAAGGTTATGGTTAGTGCC  3’-CCAGGGCTGGTCCTCCTTGG |
| KA219691 | >Unigene60679_All | 5’-CGCTCCAACCGACTCCGCAT  3’-GCTTGAGTGGGATTGGTGCT |
| KA245770 | >Unigene5849_All | 5’-TGGGTACTCGGTCTTTGTCC  3’-ATGTCCAACGGAAGAAGCAC |
| KA265299 | >Unigene25378_All | 5’-ACCTTACTGGCCTTCTCCTT  3’-CTCTGAAGGACTTGGGTTGA |

**Table 2 The general characteristics of three transcriptomes.**

| The general characteristics | | | Total Number | | |
| --- | --- | --- | --- | --- | --- |
|  |  | Samples | TW | OW | NW |
|  |  | Total Reads | 52,139,846 | 54,878,244 | 54,696,478 |
|  |  | Total Nucleotides (nt) | 4,692,586,140 | 4,939,041,960 | 4,922,683,020 |
|  |  | Q20 percentage | 98.28% | 98.23% | 98.26% |
| Unigene | Length distribution of Unigene | 100-500 nt | 51,968 | 45,149 | 50,073 |
|  |  | 500-1000 nt | 12,467 | 10,951 | 11,990 |
|  |  | 1000-1500 nt | 6,018 | 5,084 | 5,604 |
|  |  | 1500-2000 nt | 3,474 | 2,745 | 3,127 |
|  |  | >=2000 nt | 3,856 | 2,980 | 3,274 |
|  |  | N50 [1] | 1044 | 980 | 982 |
|  |  | Mean | 600 | 583 | 581 |
|  |  | All Unigene | 77,783 | 66,909 | 74,068 |
|  |  | Length of all Unigene (nt) | 46,669,068 | 38,994,548 | 43,005,342 |

**Table 3 Results of GO analysis based on biological process, cellular components and molecular functions of the three birch transcriptomes.**

| Ontology | Class | TW | OW | NW |
| --- | --- | --- | --- | --- |
| biological_process | biological adhesion | 3 | 2 | 4 |
|  | biological regulation | 1759 | 1638 | 1946 |
|  | cell killing | 2 | 1 | 2 |
|  | cellular component organization or biogenesis | 1001 | 883 | 1041 |
|  | cellular process | 7256 | 6759 | 7572 |
|  | death | 223 | 226 | 225 |
|  | developmental process | 1173 | 1111 | 1259 |
|  | establishment of localization | 1570 | 1443 | 1661 |
|  | growth | 84 | 70 | 85 |
|  | immune system process | 60 | 64 | 74 |
|  | localization | 1766 | 1604 | 1846 |
|  | locomotion | 7 | 6 | 4 |
|  | metabolic process | 7646 | 7044 | 8009 |
|  | multi-organism process | 251 | 207 | 230 |
|  | multicellular organismal process | 824 | 754 | 877 |
|  | negative regulation of biological process | 142 | 126 | 164 |
|  | nitrogen utilization | 2 | 1 | 2 |
|  | pigmentation | 4 | 4 | 1 |
|  | positive regulation of biological process | 67 | 59 | 80 |
|  | regulation of biological process | 1482 | 1398 | 1638 |
|  | reproduction | 624 | 621 | 664 |
|  | reproductive process | 620 | 620 | 663 |
|  | response to stimulus | 2352 | 2166 | 2510 |
|  | rhythmic process | 1 | 1 | 2 |
|  | signaling | 556 | 499 | 560 |
|  | viral reproduction | 12 | 8 | 9 |
| cellular_component | cell | 11248 | 10303 | 11684 |
|  | cell junction | 6 | 7 | 7 |
|  | cell part | 10048 | 9220 | 10393 |
|  | extracellular region | 21 | 6 | 16 |
|  | extracellular region part | 4 | 0 | 6 |
|  | macromolecular complex | 1076 | 965 | 1069 |
|  | membrane-enclosed lumen | 294 | 256 | 271 |
|  | organelle | 7777 | 7126 | 8057 |
|  | organelle part | 1725 | 1524 | 1650 |
|  | virion | 7 | 4 | 4 |
| molecular_function | antioxidant activity | 20 | 16 | 16 |
|  | binding | 8729 | 8132 | 8949 |
|  | catalytic activity | 8823 | 8279 | 9028 |
|  | enzyme regulator activity | 133 | 94 | 123 |
|  | molecular transducer activity | 493 | 481 | 527 |
|  | protein binding transcription factor activity | 21 | 22 | 14 |
|  | receptor activity | 59 | 51 | 56 |
|  | translation regulator activity | 1 | 4 | 1 |
|  | transporter activity | 964 | 856 | 1035 |

**Table 4 Hierarchical cluster analyses of the different family genes in response to artificial bending.**

| FLA | OW_RPKM | | | TW_RPKM | | | NW_RPKM | | T/O | | T/N | | O/N | |
| --- | --- | --- | --- | --- | --- | --- | --- | --- | --- | --- | --- | --- | --- | --- |
| Unigene51168_All | 4.2467 | | | 0.8947 | | | 0.3095 | | -2.24687 | | 1.531465 | | 3.778331 | |
| Unigene69726_All | 12.4803 | | | 4.644 | | | 49.0781 | | -1.42621 | | -3.40164 | | -1.97543 | |
| Unigene39105_All | 1.3122 | | | 0.6616 | | | 1.6478 | | -0.98796 | | -1.31651 | | -0.32855 | |
| Unigene34293_All | 2.4812 | | | 1.2827 | | | 1.8535 | | -0.95185 | | -0.53107 | | 0.420786 | |
| Unigene42901_All | 21.2135 | | | 11.3787 | | | 57.5312 | | -0.89865 | | -2.33801 | | -1.43936 | |
| Unigene10775_All | 15.3681 | | | 9.7594 | | | 24.2726 | | -0.65507 | | -1.31446 | | -0.65939 | |
| Unigene34910_All | 15.5603 | | | 16.28 | | | 26.8501 | | 0.065231 | | -0.72183 | | -0.78706 | |
| Unigene17360_All | 1052.216 | | | 1103.106 | | | 1926.769 | | 0.068141 | | -0.80461 | | -0.87275 | |
| Unigene34295_All | 16.3089 | | | 17.868 | | | 21.3079 | | 0.131719 | | -0.25401 | | -0.38573 | |
| Unigene9934_All | 895.0797 | | | 1263.896 | | | 838.3721 | | 0.497789 | | 0.592215 | | 0.094425 | |
| Unigene17441_All | 185.7818 | | | 1295.333 | | | 530.0146 | | 2.801642 | | 1.289219 | | -1.51242 | |
| Unigene20508_All | 122.2463 | | | 1345 | | | 286.0509 | | 3.459744 | | 2.233263 | | -1.22648 | |
| Unigene18144_All | 73.8013 | | | 829.1507 | | | 197.4238 | | 3.489916 | | 2.070338 | | -1.41958 | |
| Unigene26511_All | 277.4882 | | | 3492.347 | | | 791.4676 | | 3.653699 | | 2.141595 | | -1.5121 | |
| Unigene15796_All | 6.6883 | | | 85.7058 | | | 11.1429 | | 3.679681 | | 2.943268 | | -0.73641 | |
| Unigene41376_All | 36.107 | | | 500.5561 | | | 107.582 | | 3.793181 | | 2.218095 | | -1.57509 | |
| Unigene5950_All | 40.6734 | | | 743.0318 | | | 117.4633 | | 4.191266 | | 2.661214 | | -1.53005 | |
| Unigene5849_All | 67.6706 | | | 1344.095 | | | 172.483 | | 4.311962 | | 2.962109 | | -1.34985 | |
| Unigene60679_All | 242.5356 | | | 7317.225 | | | 654.6115 | | 4.915028 | | 3.482586 | | -1.43244 | |
| LTP | OW_RPKM | | | TW_RPKM | | | NW_RPKM | | T/O | | T/N | | O/N | |
| Unigene14914_All | 2.1257 | | | 0.1 | | | 1.6031 | | -4.40987 | | -4.00279 | | 0.407074 | |
| Unigene45046_All | 0.7172 | | | 0.1 | | | 4.4768 | | -2.84238 | | -5.4844 | | -2.64202 | |
| Unigene29489_All | 1.7839 | | | 0.2564 | | | 3.8318 | | -2.79857 | | -3.90155 | | -1.10299 | |
| Unigene35831_All | 15053.39 | | | 3724.332 | | | 13608.72 | | -2.01503 | | -1.86948 | | 0.145557 | |
| Unigene7651_All | 4.794 | | | 1.2086 | | | 2.8595 | | -1.98789 | | -1.24243 | | 0.745467 | |
| Unigene14712_All | 0.3756 | | | 0.1 | | | 1.1495 | | -1.9092 | | -3.52293 | | -1.61374 | |
| Unigene4323_All | 11912.6 | | | 6013.817 | | | 9884.284 | | -0.98613 | | -0.71686 | | 0.26928 | |
| Unigene41215_All | 383.6164 | | | 215.6786 | | | 461.257 | | -0.83078 | | -1.09669 | | -0.26591 | |
| Unigene71133_All | 0.4848 | | | 0.5297 | | | 7.0738 | | 0.127786 | | -3.73924 | | -3.86702 | |
| Unigene25525_All | 1.0742 | | | 2.386 | | | 1.3517 | | 1.151331 | | 0.819819 | | -0.33151 | |
| CESA | OW_RPKM | | | TW_RPKM | | | NW_RPKM | | T/O | | T/N | | O/N | |
| Unigene33109_All | 0.3531 | | | 0.0266 | | | 0.4224 | | -3.73058 | | -3.98911 | | -0.25853 | |
| Unigene37895_All | 0.146 | | | 0.0319 | | | 0.0298 | | -2.19434 | | 0.098244 | | 2.292584 | |
| Unigene33111_All | 0.4537 | | | 0.1043 | | | 0.4142 | | -2.121 | | -1.98959 | | 0.131411 | |
| Unigene26639_All | 4.6488 | | | 1.3865 | | | 13.3259 | | -1.74541 | | -3.26471 | | -1.5193 | |
| Unigene36449_All | 7.9639 | | | 2.9 | | | 4.2363 | | -1.45742 | | -0.54675 | | 0.91067 | |
| Unigene1381_All | 12.8176 | | | 6.4527 | | | 3.2018 | | -0.99015 | | 1.01102 | | 2.001171 | |
| Unigene69787_All | 3.9578 | | | 2.3634 | | | 1.5711 | | -0.74383 | | 0.589089 | | 1.332924 | |
| Unigene9574_All | 17.6261 | | | 11.2532 | | | 13.0323 | | -0.64738 | | -0.21176 | | 0.435622 | |
| Unigene42715_All | 17.9181 | | | 12.2624 | | | 17.1169 | | -0.54718 | | -0.48118 | | 0.065996 | |
| Unigene2457_All | 10.8355 | | | 8.1364 | | | 6.7165 | | -0.4133 | | 0.276681 | | 0.689984 | |
| Unigene36141_All | 5.2327 | | | 4.7789 | | | 8.2111 | | -0.13088 | | -0.7809 | | -0.65002 | |
| Unigene35422_All | 1.9423 | | | 1.8187 | | | 2.5979 | | -0.09486 | | -0.51444 | | -0.41958 | |
| Unigene19463_All | 21.3273 | | | 20.745 | | | 22.6895 | | -0.03994 | | -0.12926 | | -0.08932 | |
| Unigene4392_All | 3.0691 | | | 3.0916 | | | 5.0834 | | 0.010538 | | -0.71744 | | -0.72798 | |
| Unigene43083_All | 9.272 | | | 9.3759 | | | 6.7191 | | 0.016077 | | 0.480689 | | 0.464613 | |
| Unigene2619_All | 2.1125 | | | 2.1389 | | | 3.8639 | | 0.017918 | | -0.85319 | | -0.87111 | |
| Unigene4926_All | 1.1497 | | | 1.1694 | | | 3.0337 | | 0.024511 | | -1.37531 | | -1.39982 | |
| Unigene25981_All | 40.3158 | | | 41.2751 | | | 43.1782 | | 0.033926 | | -0.06503 | | -0.09896 | |
| Unigene1382_All | 26.9524 | | | 30.5287 | | | 42.5646 | | 0.179752 | | -0.47949 | | -0.65924 | |
| Unigene25301_All | 34.9747 | | | 40.8382 | | | 53.6006 | | 0.223608 | | -0.39233 | | -0.61594 | |
| Unigene25699_All | 87.0543 | | | 102.0784 | | | 104.5198 | | 0.22969 | | -0.0341 | | -0.26379 | |
| Unigene6922_All | 22.1948 | | | 28.4142 | | | 19.9713 | | 0.35639 | | 0.508684 | | 0.152293 | |
| Unigene10121_All | 7.5133 | | | 9.9404 | | | 7.6449 | | 0.403857 | | 0.378806 | | -0.02505 | |
| Unigene10028_All | 73.8519 | | | 99.1735 | | | 95.7427 | | 0.42532 | | 0.050792 | | -0.37453 | |
| Unigene3305_All | 31.0018 | | | 44.502 | | | 38.1392 | | 0.521518 | | 0.222596 | | -0.29892 | |
| Unigene1015_All | 12.1256 | | | 21.2389 | | | 19.6041 | | 0.808653 | | 0.115554 | | -0.6931 | |
| Unigene72323_All | 4.8574 | | | 9.0723 | | | 5.9551 | | 0.901284 | | 0.607343 | | -0.29394 | |
| Unigene1383_All | 0.9855 | | | 1.9776 | | | 1.0383 | | 1.004823 | | 0.929527 | | -0.0753 | |
| Unigene29774_All | 9.818 | | | 19.8622 | | | 9.5534 | | 1.016524 | | 1.055939 | | 0.039415 | |
| Unigene33113_All | 0.8382 | | | 2.0904 | | | 0.4147 | | 1.318413 | | 2.333639 | | 1.015226 | |
| Unigene48862_All | 18.1513 | | | 56.8881 | | | 17.6889 | | 1.648054 | | 1.685283 | | 0.037229 | |
| Unigene1016_All | 1.2056 | | | 3.8094 | | | 1.7221 | | 1.659812 | | 1.145395 | | -0.51442 | |
| Unigene26227_All | 325.4991 | | | 1031.166 | | | 521.6003 | | 1.663551 | | 0.983259 | | -0.68029 | |
| Unigene58742_All | 1.3243 | | | 4.5008 | | | 0.0751 | | 1.764951 | | 5.905225 | | 4.140273 | |
| Unigene16894_All | 30.8717 | | | 325.6248 | | | 51.2644 | | 3.398854 | | 2.667181 | | -0.73167 | |
| Unigene40841_All | 0.6241 | | | 8.5034 | | | 1.2381 | | 3.768191 | | 2.779912 | | -0.98828 | |
| Unigene69238_All | 0.2869 | | | 5.1716 | | | 0.1952 | | 4.171991 | | 4.727586 | | 0.555595 | |
| Unigene16893_All | 3.3076 | | | 71.1455 | | | 9.3363 | | 4.426916 | | 2.92985 | | -1.49707 | |
| Unigene68728_All | 0.0747 | | | 3.182 | | | 0.4572 | | 5.412682 | | 2.799037 | | -2.61365 | |
| SuSy | OW_RPKM | | | TW_RPKM | | | NW_RPKM | | T/O | | T/N | | O/N | |
| Unigene34644_All | 0.4821 | | | 1.275 | | | 0.5522 | | 1.403093 | | 1.207234 | | -0.19586 | |
| Unigene34645_All | 8.4945 | | | 32.6865 | | | 17.0165 | | 1.944094 | | 0.941761 | | -1.00233 | |
| Unigene10208_All | 62.6399 | | | 104.9286 | | | 54.4581 | | 0.744254 | | 0.946189 | | 0.201935 | |
| Unigene70265_All | 32.3148 | | | 51.8214 | | | 99.9928 | | 0.681353 | | -0.94828 | | -1.62963 | |
| Unigene43572_All | 2.6968 | | | 4.2228 | | | 2.3847 | | 0.646951 | | 0.824392 | | 0.177441 | |
| SPS | OW_RPKM | | | TW_RPKM | | | NW_RPKM | | T/O | | T/N | | O/N | |
| Unigene58406_All | 0.6572 | | | 4.0387 | | | 0.0838 | | 2.619487 | | 5.590797 | | 2.97131 | |
| Unigene46551_All | 0.4796 | | | 3.6675 | | | 1.3864 | | 2.934893 | | 1.403453 | | -1.53144 | |
| Unigene11933_All | 0.1573 | | | 8.421 | | | 1.7656 | | 5.742401 | | 2.253833 | | -3.48857 | |
| Unigene41633_All | 2.9759 | | | 8.8482 | | | 3.5684 | | 1.572058 | | 1.310107 | | -0.26195 | |
| Unigene4846_All | 13.1782 | | | 40.7485 | | | 17.2319 | | 1.628594 | | 1.241665 | | -0.38693 | |
| Unigene42290_All | 80.7052 | | | 83.8264 | | | 73.0556 | | 0.054743 | | 0.19841 | | 0.143667 | |
| GALE | OW_RPKM | | | TW_RPKM | | | NW_RPKM | | T/O | | T/N | | O/N | |
| Unigene10766 | 24.1664 | | | 18.4021 | | | 28.1057 | | -0.39313 | | -0.61099 | | -0.21786 | |
| Unigene38450e | 5.0913 | | | 25.6846 | | | 7.0951 | | 2.334798 | | 1.856009 | | -0.47879 | |
| Unigene68264 | 0.1 | | | 2.5905 | | | 0.8065 | | 4.695159 | | 1.683484 | | -3.01167 | |
| UTP-glucose 1 phosphate  uridylyltransferase | OW_RPKM | | TW_RPKM | | | NW_RPKM | | | T/O | | T/N | | O/N | |
| Unigene1654_All | | 11.1698 | | | 13.0662 | | | 16.8021 | | 0.226236 | | -0.3628 | | -0.58904 |
| Unigene17365_All | | 63.8964 | | | 96.1162 | | | 66.5668 | | 0.589045 | | 0.529977 | | -0.05907 |
| Unigene17366_All | | 3.7864 | | | 3.0266 | | | 3.1094 | | -0.32313 | | -0.03894 | | 0.284191 |
| Unigene25992_All | | 88.035 | | | 191.6233 | | | 90.7054 | | 1.122124 | | 1.079013 | | -0.04311 |
| Unigene48266_All | | 2.7744 | | | 13.7602 | | | 6.2841 | | 2.310254 | | 1.130723 | | -1.17953 |
| Unigene67536_All | | 0.0677 | | | 0.02 | | | 0.02 | | -1.75916 | | 0 | | 1.759156 |
| EXP | | OW_RPKM | | | TW_RPKM | | | NW_RPKM | | T/O | | T/N | | O/N |
| Unigene26983_All | | 21.4659 | | | 22.029 | | | 49.997 | | 0.037357 | | -1.18244 | | -1.21979 |
| Unigene31009_All | | 3.0722 | | | 10.3995 | | | 1.5452 | | 1.75917 | | 2.750649 | | 0.991479 |
| Unigene36216_All | | 54.5262 | | | 92.381 | | | 111.0497 | | 0.760647 | | -0.26554 | | -1.02618 |
| Unigene43047_All | | 12.3408 | | | 21.7913 | | | 15.9559 | | 0.820316 | | 0.449662 | | -0.37065 |
| Unigene43581_All | | 18.5496 | | | 31.5338 | | | 42.2935 | | 0.765511 | | -0.42354 | | -1.18905 |
| Unigene54685_All | | 8.2907 | | | 7.3126 | | | 25.6221 | | -0.18111 | | -1.80893 | | -1.62782 |
| Unigene80094_All | | 0.4382 | | | 0.0957 | | | 2.6375 | | -2.195 | | -4.78451 | | -2.58951 |
| DHQ-SDH | | OW_RPKM | | | TW_RPKM | | | NW_RPKM | | T/O | | T/N | | O/N |
| Unigene79883_All | | 2.6585 | | | 0.3267 | | | 16.0708 | | -3.02457 | | -5.62033 | | -2.59576 |
| Unigene34167_All | | 3.1508 | | | 0.8694 | | | 1.9388 | | -1.85763 | | -1.15707 | | 0.700554 |
| Unigene47317_All | | 1.2708 | | | 0.3786 | | | 0.943 | | -1.74699 | | -1.31658 | | 0.430407 |
| Unigene164_All | | 0.1987 | | | 0.0592 | | | 0.0737 | | -1.74692 | | -0.31607 | | 1.430855 |
| Unigene171_All | | 2.1815 | | | 0.8171 | | | 0.0636 | | -1.41674 | | 3.683414 | | 5.10015 |
| Unigene51233_All | | 0.7116 | | | 0.2915 | | | 0.1 | | -1.28757 | | 1.543496 | | 2.831067 |
| Unigene165_All | | 0.5177 | | | 0.2545 | | | 0.8803 | | -1.02445 | | -1.79033 | | -0.76588 |
| Unigene26699_All | | 74.9562 | | | 72.0084 | | | 57.2756 | | -0.05788 | | 0.330245 | | 0.388127 |
| Unigene17737_All | | 4.902 | | | 6.8947 | | | 4.8139 | | 0.492117 | | 0.518282 | | 0.026164 |
| Unigene5892_All | | 17.0993 | | | 24.1814 | | | 6.3572 | | 0.499961 | | 1.927434 | | 1.427474 |
| Unigene79993_All | | 4.8195 | | | 7.6792 | | | 11.1298 | | 0.672073 | | -0.5354 | | -1.20747 |
| Unigene17117_All | | 1.9348 | | | 3.2784 | | | 2.5381 | | 0.760807 | | 0.369243 | | -0.39156 |
| Unigene17116_All | | 0.512 | | | 1.0325 | | | 2.4309 | | 1.011926 | | -1.23535 | | -2.24727 |
| Unigene17118_All | | 1.2648 | | | 4.1772 | | | 3.4662 | | 1.723627 | | 0.269181 | | -1.45445 |
| SK | | OW_RPKM | | | TW_RPKM | | | NW_RPKM | | T/O | | T/N | | O/N |
| Unigene5195_All | | 6.0853 | | | 5.0904 | | | 3.799 | | -0.25755 | | 0.422159 | | 0.679709 |
| Unigene37687_All | | 1.3196 | | | 1.1908 | | | 0.9951 | | -0.14817 | | 0.259018 | | 0.407187 |
| Unigene54946_All | | 0.2694 | | | 0.0589 | | | 0.055 | | -2.19341 | | 0.098836 | | 2.292246 |
| Unigene60180_All | | 0.7203 | | | 0.8852 | | | 0.1 | | 0.297406 | | 3.146003 | | 2.848598 |
| Unigene41944_All | | 8.3877 | | | 8.5857 | | | 8.7688 | | 0.033661 | | -0.03044 | | -0.0641 |
| Unigene44668_All | | 4.6794 | | | 3.4945 | | | 4.2708 | | -0.42124 | | -0.28942 | | 0.131817 |
| Unigene25578_All | | 4.0893 | | | 4.3771 | | | 2.2336 | | 0.098121 | | 0.970604 | | 0.872483 |
| Unigene22217_All | | 15.4007 | | | 16.36 | | | 15.9466 | | 0.087177 | | 0.036924 | | -0.05025 |
| Unigene16056_All | | 0.8348 | | | 0.1303 | | | 0.6388 | | -2.67959 | | -2.29353 | | 0.386066 |
| EPSP | | OW_RPKM | | | TW_RPKM | | | NW_RPKM | | T/O | | T/N | | O/N |
| Unigene42889_All | | 1.5294 | | | 0.4386 | | | 2.8673 | | -1.80199 | | -2.70872 | | -0.90673 |
| Unigene45671_All | | 46.3764 | | | 42.5935 | | | 23.7487 | | -0.12276 | | 0.842785 | | 0.965542 |
| Unigene69843_All | | 61.0758 | | | 33.8637 | | | 100.4747 | | -0.85086 | | -1.56902 | | -0.71816 |
| PAL | | OW_RPKM | | | TW_RPKM | | | NW_RPKM | | T/O | | T/N | | O/N |
| Unigene10584_All | | 42.1443 | | | 48.4754 | | | 63.2097 | | 0.201915 | | -0.38289 | | -0.58481 |
| Unigene27073_All | | 89.8502 | | | 14.7387 | | | 26.6064 | | -2.60791 | | -0.85216 | | 1.755748 |
| Unigene33308_All | | 146.0789 | | | 23.5269 | | | 35.6293 | | -2.63436 | | -0.59875 | | 2.035612 |
| Unigene37987_All | | 204.0541 | | | 34.7663 | | | 50.3271 | | -2.55319 | | -0.53365 | | 2.019544 |
| Unigene3963_All | | 78.6667 | | | 109.9567 | | | 92.5904 | | 0.483111 | | 0.248001 | | -0.23511 |
| Unigene3964_All | | 81.5748 | | | 52.8873 | | | 114.4192 | | -0.6252 | | -1.11334 | | -0.48813 |
| Unigene41537_All | | 7.0671 | | | 13.2267 | | | 4.4079 | | 0.904263 | | 1.58529 | | 0.681027 |
| Unigene43286_All | | 21.1117 | | | 28.7581 | | | 25.1218 | | 0.445926 | | 0.195029 | | -0.2509 |
| Unigene50727_All | | 2.3376 | | | 0.3331 | | | 0.6222 | | -2.811 | | -0.90142 | | 1.909578 |
| COMT | | OW_RPKM | | | TW_RPKM | | | NW_RPKM | | T/O | | T/N | | O/N |
| Unigene25260_All | | 21.3135 | | | 18.1564 | | | 21.9426 | | -0.23129 | | -0.27326 | | -0.04197 |
| Unigene43973_All | | 73.7824 | | | 144.6658 | | | 84.5507 | | 0.971375 | | 0.774835 | | -0.19654 |
| Unigene59656_All | | 0.1314 | | | 2.5847 | | | 0.2682 | | 4.29796 | | 3.268616 | | -1.02934 |
| Unigene6508_All | | 22.5336 | | | 11.4853 | | | 12.3641 | | -0.97229 | | -0.10637 | | 0.865921 |
| Unigene73959_All | | 83.5416 | | | 69.655 | | | 172.7167 | | -0.26227 | | -1.31011 | | -1.04784 |
| 4CL | | OW_RPKM | | | TW_RPKM | | | NW_RPKM | | T/O | | T/N | | O/N |
| Unigene41938_All | | 4.1715 | | | 0.1 | | | 2.2954 | | -5.38249 | | -4.52067 | | 0.861821 |
| Unigene22253_All | | 4.3659 | | | 0.4616 | | | 1.7243 | | -3.24156 | | -1.9013 | | 1.340268 |
| Unigene7381_All | | 6.9199 | | | 0.8284 | | | 1.8376 | | -3.06235 | | -1.14942 | | 1.912928 |
| Unigene48606_All | | 1.2497 | | | 0.4551 | | | 2.6917 | | -1.45733 | | -2.56426 | | -1.10694 |
| Unigene75018_All | | 4.1707 | | | 1.5963 | | | 4.7522 | | -1.38556 | | -1.57386 | | -0.18831 |
| Unigene9542_All | | 6.4329 | | | 2.6961 | | | 2.9308 | | -1.2546 | | -0.12042 | | 1.134175 |
| Unigene23316_All | | 5.5251 | | | 2.3966 | | | 2.0725 | | -1.20501 | | 0.209617 | | 1.414628 |
| Unigene11924_All | | 13.3589 | | | 7.9545 | | | 2.3974 | | -0.74796 | | 1.730301 | | 2.478259 |
| Unigene37622_All | | 6.783 | | | 4.1034 | | | 5.1718 | | -0.7251 | | -0.33385 | | 0.391257 |
| Unigene35414_All | | 4.095 | | | 2.7339 | | | 3.2961 | | -0.5829 | | -0.2698 | | 0.313103 |
| Unigene43275_All | | 2.837 | | | 1.937 | | | 4.0523 | | -0.55054 | | -1.06492 | | -0.51437 |
| Unigene23547_All | | 1.3145 | | | 0.9389 | | | 0.5158 | | -0.48547 | | 0.86416 | | 1.34963 |
| Unigene21960_All | | 18.6019 | | | 22.6293 | | | 18.7846 | | 0.282742 | | 0.268642 | | -0.0141 |
| Unigene12617_All | | 0.9206 | | | 1.76 | | | 1.409 | | 0.934929 | | 0.320904 | | -0.61403 |
| Unigene68289_All | | 0.1 | | | 1.2022 | | | 0.5614 | | 3.587605 | | 1.098576 | | -2.48903 |
| Unigene57851_All | | 0.1 | | | 1.9581 | | | 0.3325 | | 4.291383 | | 2.558028 | | -1.73335 |
| CCoAOMT | | OW_RPKM | | | TW_RPKM | | | NW_RPKM | | T/O | | T/N | | O/N |
| Unigene80685_All | | 0.0739 | | | 0.01 | | | 2.7158 | | -2.88557 | | -8.08523 | | -5.19966 |
| Unigene4853_All | | 122.42 | | | 28.2471 | | | 57.0235 | | -2.11566 | | -1.01345 | | 1.102211 |
| Unigene75610_All | | 0.3669 | | | 0.1 | | | 2.1714 | | -1.87539 | | -4.44055 | | -2.56517 |
| Unigene26427_All | | 234.82 | | | 211.4524 | | | 289.246 | | -0.15122 | | -0.45196 | | -0.30074 |
| Unigene5290_All | | 1.1686 | | | 2.0868 | | | 1.7426 | | 0.836511 | | 0.260051 | | -0.57646 |
| Unigene53546_All | | 3.9108 | | | 9.227 | | | 0.9144 | | 1.238398 | | 3.334964 | | 2.096566 |
| Unigene46448_All | | 1.0733 | | | 3.6044 | | | 0.8923 | | 1.747706 | | 2.014158 | | 0.266453 |
| Unigene67505_All | | 0.0758 | | | 0.29 | | | 0.01 | | 1.935783 | | 4.857981 | | 2.922198 |
| CCR | | OW_RPKM | | | TW_RPKM | | | NW_RPKM | | T/O | | T/N | | O/N |
| Unigene69778_All | | 1.4594 | | | 0.1 | | | 8.712 | | -3.8673 | | -6.44493 | | -2.57763 |
| Unigene18177_All | | 0.306 | | | 0.0478 | | | 0.0446 | | -2.67845 | | 0.099967 | | 2.778416 |
| Unigene9207_All | | 4.6459 | | | 1.795 | | | 1.1561 | | -1.37197 | | 0.634718 | | 2.006692 |
| Unigene48778_All | | 2.367 | | | 1.6876 | | | 1.4236 | | -0.48809 | | 0.245429 | | 0.733516 |
| Unigene77365_All | | 0.8399 | | | 0.7209 | | | 1.6526 | | -0.22042 | | -1.19687 | | -0.97645 |
| Unigene14857_All | | 7.4273 | | | 6.6519 | | | 6.076 | | -0.15907 | | 0.130645 | | 0.289716 |
| Unigene12921_All | | 2.3212 | | | 2.0799 | | | 1.0112 | | -0.15836 | | 1.040446 | | 1.198802 |
| Unigene31965_All | | 2.3585 | | | 2.2289 | | | 2.2727 | | -0.08154 | | -0.02808 | | 0.053462 |
| Unigene10747_All | | 527.2071 | | | 580.8283 | | | 559.2649 | | 0.139742 | | 0.05458 | | -0.08516 |
| Unigene55781_All | | 0.5225 | | | 0.6089 | | | 0.1422 | | 0.220774 | | 2.098284 | | 1.87751 |
| Unigene25489_All | | 110.6667 | | | 131.992 | | | 120.2094 | | 0.254229 | | 0.134901 | | -0.11933 |
| Unigene36409_All | | 6.6289 | | | 9.6026 | | | 6.0697 | | 0.534656 | | 0.6618 | | 0.127144 |
| Unigene69777_All | | 0.0947 | | | 0.138 | | | 0.01 | | 0.543232 | | 3.786596 | | 3.243364 |
| Unigene47497_All | | 0.5377 | | | 0.9791 | | | 2.7432 | | 0.864655 | | -1.48633 | | -2.35099 |
| Unigene18176_All | | 1.2539 | | | 2.3146 | | | 1.9411 | | 0.884341 | | 0.253888 | | -0.63045 |
| Unigene18179_All | | 3.9967 | | | 8.6378 | | | 5.2459 | | 1.111855 | | 0.719474 | | -0.39238 |
| Unigene58649_All | | 5.6398 | | | 12.692 | | | 4.051 | | 1.170204 | | 1.647569 | | 0.477366 |
| Unigene29770_All | | 0.2202 | | | 3.287 | | | 0.7862 | | 3.899885 | | 2.063803 | | -1.83608 |
| CAD | | OW_RPKM | | | TW_RPKM | | | NW_RPKM | | T/O | | T/N | | O/N |
| Unigene69778_All | | 1.4594 | | | 0.1 | | | 8.712 | | -3.8673 | | -6.44493 | | -2.57763 |
| Unigene23748_All | | 2.5814 | | | 0.564 | | | 5.3427 | | -2.19439 | | -3.2438 | | -1.04942 |
| Unigene33432_All | | 7.7851 | | | 2.1262 | | | 0.389 | | -1.87244 | | 2.450435 | | 4.322874 |
| Unigene33433_All | | 7.7342 | | | 3.3392 | | | 1.2002 | | -1.21175 | | 1.476228 | | 2.687977 |
| Unigene26430_All | | 64.5698 | | | 39.0443 | | | 74.8559 | | -0.72575 | | -0.939 | | -0.21326 |
| Unigene79431_All | | 0.7482 | | | 0.5048 | | | 0.6736 | | -0.56771 | | -0.41618 | | 0.151532 |
| Unigene44271_All | | 3.1324 | | | 2.2282 | | | 8.7701 | | -0.49139 | | -1.97671 | | -1.48532 |
| Unigene33431_All | | 0.819 | | | 0.6913 | | | 0.2279 | | -0.24455 | | 1.600911 | | 1.845463 |
| Unigene74005_All | | 7.4334 | | | 6.3063 | | | 24.6484 | | -0.23723 | | -1.96663 | | -1.7294 |
| Unigene42808_All | | 205.8911 | | | 221.3531 | | | 212.3241 | | 0.104468 | | 0.060081 | | -0.04439 |
| Unigene39608_All | | 2.9014 | | | 3.3741 | | | 2.0371 | | 0.217754 | | 0.727986 | | 0.510232 |
| Unigene69777_All | | 0.0947 | | | 0.138 | | | 0.01 | | 0.543232 | | 3.786596 | | 3.243364 |
| Unigene44215_All | | 81.7162 | | | 140.5914 | | | 152.3203 | | 0.782814 | | -0.1156 | | -0.89841 |
| Unigene69618_All | | 77.418 | | | 135.7856 | | | 89.8536 | | 0.81059 | | 0.595682 | | -0.21491 |
| Unigene20180_All | | 10.6604 | | | 19.5061 | | | 10.6739 | | 0.871664 | | 0.869838 | | -0.00183 |
| Unigene62955_All | | 0.1 | | | 0.7153 | | | 0.4454 | | 2.838548 | | 0.683447 | | -2.1551 |
| POD | | OW_RPKM | | | TW_RPKM | | | NW_RPKM | | T/O | | T/N | | O/N |
| Unigene13850_All | | 3.6796 | | | 0.1 | | | 1.4167 | | -5.20148 | | -3.82446 | | 1.377015 |
| Unigene49994_All | | 2.5321 | | | 0.1106 | | | 3.5135 | | -4.51691 | | -4.98949 | | -0.47257 |
| Unigene48933_All | | 3.6019 | | | 0.361 | | | 2.3601 | | -3.31869 | | -2.70878 | | 0.60991 |
| Unigene18727_All | | 5.2981 | | | 0.7326 | | | 3.6949 | | -2.85438 | | -2.33444 | | 0.51994 |
| Unigene41200_All | | 4.4053 | | | 0.6266 | | | 2.9496 | | -2.81362 | | -2.2349 | | 0.578721 |
| Unigene31049_All | | 2.7977 | | | 0.4366 | | | 1.6311 | | -2.67986 | | -1.90146 | | 0.778396 |
| Unigene18728_All | | 6.5635 | | | 1.2958 | | | 4.8409 | | -2.34062 | | -1.90143 | | 0.43919 |
| Unigene14894_All | | 1.0978 | | | 0.2351 | | | 1.4055 | | -2.22327 | | -2.57974 | | -0.35647 |
| Unigene18729_All | | 7.0194 | | | 1.5401 | | | 4.0453 | | -2.18832 | | -1.39322 | | 0.795101 |
| Unigene14188_All | | 2.2233 | | | 0.5113 | | | 1.9102 | | -2.12046 | | -1.90148 | | 0.218979 |
| Unigene31477_All | | 1.8277 | | | 0.4538 | | | 0.9324 | | -2.0099 | | -1.03889 | | 0.971008 |
| Unigene28422_All | | 1.023 | | | 0.3048 | | | 1.5182 | | -1.74687 | | -2.31643 | | -0.56956 |
| Unigene28422_All | | 1.023 | | | 0.3048 | | | 1.5182 | | -1.74687 | | -2.31643 | | -0.56956 |
| Unigene31212_All | | 1.2945 | | | 0.5439 | | | 2.3368 | | -1.25098 | | -2.10312 | | -0.85214 |
| Unigene35681_All | | 0.2428 | | | 0.1206 | | | 0.0676 | | -1.00954 | | 0.835135 | | 1.844673 |
| Unigene40686_All | | 4.3707 | | | 2.2282 | | | 1.9324 | | -0.97199 | | 0.205485 | | 1.177471 |
| Unigene21838_All | | 1.5718 | | | 0.8586 | | | 7.7232 | | -0.87236 | | -3.16914 | | -2.29678 |
| Unigene15153_All | | 17.3771 | | | 11.4555 | | | 6.332 | | -0.60115 | | 0.855307 | | 1.456454 |
| Unigene39834_All | | 1.4083 | | | 0.9402 | | | 3.6721 | | -0.58292 | | -1.96557 | | -1.38265 |
| Unigene15798_All | | 0.6406 | | | 0.4666 | | | 1.9608 | | -0.45724 | | -2.07118 | | -1.61395 |
| Unigene26530_All | | 63.4115 | | | 51.8989 | | | 139.7403 | | -0.28904 | | -1.42897 | | -1.13993 |
| Unigene10463_All | | 15.1704 | | | 13.1771 | | | 42.2216 | | -0.20323 | | -1.67995 | | -1.47672 |
| Unigene5094_All | | 8.0448 | | | 8.5337 | | | 3.4497 | | 0.085115 | | 1.3067 | | 1.221586 |
| Unigene37906_All | | 9.9335 | | | 14.8135 | | | 6.5498 | | 0.576539 | | 1.17739 | | 0.600851 |
| Unigene20442_All | | 6.5774 | | | 10.6776 | | | 3.3789 | | 0.698998 | | 1.659962 | | 0.960964 |
| Unigene65025_All | | 0.8531 | | | 1.398 | | | 0.2901 | | 0.712578 | | 2.268742 | | 1.556165 |
| Unigene22391_All | | 0.4219 | | | 0.6914 | | | 4.8642 | | 0.71262 | | -2.81461 | | -3.52723 |
| Unigene60683_All | | 2.1962 | | | 5.2782 | | | 8.9629 | | 1.265037 | | -0.76392 | | -2.02896 |
| Unigene5774_All | | 2.6465 | | | 7.0043 | | | 6.7504 | | 1.404155 | | 0.053268 | | -1.35089 |
| Unigene15514_All | | 0.4362 | | | 2.621 | | | 2.6259 | | 2.587056 | | -0.00269 | | -2.58975 |
| Unigene58735_All | | 0.0885 | | | 0.7975 | | | 0.4514 | | 3.171735 | | 0.821078 | | -2.35066 |
| Unigene62814_All | | 0.1 | | | 1.2629 | | | 0.5897 | | 3.658669 | | 1.098687 | | -2.55998 |
| Unigene45922_All | | 2.7211 | | | 61.3291 | | | 1.866 | | 4.49431 | | 5.038551 | | 0.544241 |
| MYB | | OW_RPKM | | | TW_RPKM | | | NW_RPKM | | T/O | | T/N | | O/N |
| Unigene35498_All | | 48.0877 | | | 3.4607 | | | 17.5243 | | -3.79653 | | -2.34022 | | 1.456311 |
| Unigene2586_All | | 0.4336 | | | 0.0338 | | | 0.2212 | | -3.68127 | | -2.71026 | | 0.971013 |
| Unigene52230_All | | 1.195 | | | 0.1 | | | 0.1524 | | -3.57894 | | -0.60786 | | 2.971076 |
| Unigene19087_All | | 2.6766 | | | 0.3185 | | | 2.5416 | | -3.07104 | | -2.99637 | | 0.074665 |
| Unigene19088_All | | 40.3898 | | | 5.1453 | | | 30.9269 | | -2.97266 | | -2.58754 | | 0.385129 |
| Unigene44233_All | | 1.7737 | | | 0.2704 | | | 3.7456 | | -2.71359 | | -3.79203 | | -1.07843 |
| Unigene7298_All | | 5.2614 | | | 1.045 | | | 9.2722 | | -2.33194 | | -3.14941 | | -0.81746 |
| Unigene6411_All | | 9.93 | | | 2.1403 | | | 12.7601 | | -2.21398 | | -2.57575 | | -0.36177 |
| Unigene3836_All | | 50.6865 | | | 12.49 | | | 11.5312 | | -2.02083 | | 0.115231 | | 2.136059 |
| Unigene27461_All | | 11.2895 | | | 3.0688 | | | 6.8137 | | -1.87924 | | -1.15076 | | 0.728471 |
| Unigene11071_All | | 7.4907 | | | 2.1869 | | | 5.8637 | | -1.77621 | | -1.42292 | | 0.353289 |
| Unigene42934_All | | 2.8602 | | | 0.902 | | | 13.0869 | | -1.66492 | | -3.85885 | | -2.19394 |
| Unigene41157_All | | 64.4274 | | | 20.8349 | | | 71.2975 | | -1.62867 | | -1.77485 | | -0.14618 |
| Unigene55769_All | | 6.6042 | | | 2.2854 | | | 0.9207 | | -1.53094 | | 1.311644 | | 2.842581 |
| Unigene9213_All | | 103.8742 | | | 37.6416 | | | 39.2768 | | -1.46444 | | -0.06135 | | 1.403088 |
| Unigene35166_All | | 5.3639 | | | 2.016 | | | 9.4392 | | -1.41179 | | -2.22717 | | -0.81538 |
| Unigene23251_All | | 2.0343 | | | 0.7693 | | | 4.4704 | | -1.40291 | | -2.53879 | | -1.13587 |
| Unigene39404_All | | 9.8943 | | | 4.3471 | | | 4.1148 | | -1.18654 | | 0.079231 | | 1.265775 |
| Unigene20778_All | | 26.592 | | | 11.8285 | | | 72.9581 | | -1.16873 | | -2.6248 | | -1.45608 |
| Unigene42165_All | | 0.7358 | | | 0.332 | | | 0.457 | | -1.14813 | | -0.46101 | | 0.68712 |
| Unigene14776_All | | 1.885 | | | 0.8521 | | | 4.8413 | | -1.14547 | | -2.5063 | | -1.36083 |
| Unigene3249_All | | 2.4953 | | | 1.2051 | | | 3.591 | | -1.05006 | | -1.57523 | | -0.52517 |
| Unigene4576_All | | 436.4255 | | | 213.3987 | | | 172.227 | | -1.03218 | | 0.30924 | | 1.341424 |
| Unigene2383_All | | 2.2781 | | | 1.1192 | | | 0.6877 | | -1.02536 | | 0.702617 | | 1.72798 |
| Unigene32701_All | | 0.2278 | | | 0.1244 | | | 0.0194 | | -0.87278 | | 2.680858 | | 3.553639 |
| Unigene389_All | | 0.0862 | | | 0.0471 | | | 0.1 | | -0.87196 | | -1.0862 | | -0.21424 |
| Unigene26560_All | | 3.2091 | | | 1.8138 | | | 7.193 | | -0.82315 | | -1.98758 | | -1.16442 |
| Unigene5057_All | | 2.8068 | | | 1.5899 | | | 1.3258 | | -0.81999 | | 0.262073 | | 1.082063 |
| Unigene7006_All | | 2.2128 | | | 1.3017 | | | 4.3417 | | -0.76548 | | -1.73786 | | -0.97239 |
| Unigene25179_All | | 5.4316 | | | 3.2603 | | | 22.7151 | | -0.73637 | | -2.80058 | | -2.0642 |
| Unigene18653_All | | 119.0805 | | | 77.9284 | | | 89.8467 | | -0.61172 | | -0.20532 | | 0.4064 |
| Unigene32696_All | | 0.1613 | | | 0.1058 | | | 0.6585 | | -0.60841 | | -2.63784 | | -2.02944 |
| Unigene19514_All | | 3.1819 | | | 2.1837 | | | 9.0734 | | -0.54311 | | -2.05487 | | -1.51175 |
| Unigene18654_All | | 29.913 | | | 21.1995 | | | 34.0053 | | -0.49674 | | -0.68173 | | -0.18499 |
| Unigene10649_All | | 24.2816 | | | 17.406 | | | 24.2074 | | -0.48028 | | -0.47586 | | 0.004415 |
| Unigene1531_All | | 0.5297 | | | 0.3858 | | | 3.4229 | | -0.45732 | | -3.14929 | | -2.69197 |
| Unigene33213_All | | 1.4542 | | | 1.0964 | | | 1.3792 | | -0.40745 | | -0.33106 | | 0.076394 |
| Unigene48233_All | | 26.7456 | | | 21.3899 | | | 47.6144 | | -0.32237 | | -1.15447 | | -0.8321 |
| Unigene3980_All | | 2.332 | | | 1.9005 | | | 3.2479 | | -0.29519 | | -0.77313 | | -0.47794 |
| Unigene19513_All | | 6.6497 | | | 5.4656 | | | 11.2753 | | -0.28291 | | -1.04471 | | -0.7618 |
| Unigene4208_All | | 10.5407 | | | 8.776 | | | 19.4729 | | -0.26434 | | -1.14983 | | -0.8855 |
| Unigene9098_All | | 9.7645 | | | 8.144 | | | 7.3378 | | -0.26181 | | 0.15039 | | 0.412199 |
| Unigene29871_All | | 0.9363 | | | 0.7822 | | | 0.4495 | | -0.25943 | | 0.799216 | | 1.05865 |
| Unigene20859_All | | 0.5318 | | | 0.4469 | | | 0.0835 | | -0.25093 | | 2.420104 | | 2.671036 |
| Unigene41261_All | | 13.5248 | | | 11.6153 | | | 14.381 | | -0.21958 | | -0.30814 | | -0.08856 |
| Unigene9936_All | | 37.3225 | | | 32.2941 | | | 28.648 | | -0.20878 | | 0.172836 | | 0.381611 |
| Unigene15775_All | | 1.4555 | | | 1.272 | | | 1.782 | | -0.19442 | | -0.4864 | | -0.29198 |
| Unigene3032_All | | 117.8545 | | | 104.7854 | | | 184.173 | | -0.16957 | | -0.81363 | | -0.64406 |
| Unigene5619_All | | 5.5221 | | | 4.9305 | | | 4.659 | | -0.16348 | | 0.081714 | | 0.245197 |
| Unigene38791_All | | 4.2143 | | | 3.7817 | | | 1.3053 | | -0.15626 | | 1.534653 | | 1.690912 |
| Unigene22234_All | | 84.3965 | | | 76.3328 | | | 82.6229 | | -0.14488 | | -0.11424 | | 0.030641 |
| Unigene4103_All | | 2.366 | | | 2.1539 | | | 7.0409 | | -0.1355 | | -1.70881 | | -1.57331 |
| Unigene18346_All | | 2.0694 | | | 1.8839 | | | 1.9354 | | -0.13549 | | -0.03891 | | 0.096581 |
| Unigene41277_All | | 8.5558 | | | 8.1177 | | | 7.4909 | | -0.07583 | | 0.115932 | | 0.191764 |
| Unigene17690_All | | 21.2215 | | | 20.4138 | | | 23.3762 | | -0.05598 | | -0.1955 | | -0.13951 |
| Unigene17416_All | | 5.4521 | | | 5.2462 | | | 7.5826 | | -0.05554 | | -0.53142 | | -0.47588 |
| Unigene17934_All | | 0.6926 | | | 0.6725 | | | 2.748 | | -0.04249 | | -2.03078 | | -1.98829 |
| Unigene7875_All | | 21.3809 | | | 21.1965 | | | 34.2113 | | -0.0125 | | -0.69065 | | -0.67815 |
| Unigene33212_All | | 0.6801 | | | 0.6755 | | | 1.2196 | | -0.00979 | | -0.85238 | | -0.84259 |
| Unigene41731_All | | 6.0841 | | | 6.0926 | | | 9.2373 | | 0.002014 | | -0.60041 | | -0.60243 |
| Unigene30510_All | | 1.0269 | | | 1.0471 | | | 4.2608 | | 0.028104 | | -2.02473 | | -2.05283 |
| Unigene6573_All | | 39.2037 | | | 40.2045 | | | 36.2034 | | 0.036367 | | 0.151232 | | 0.114865 |
| Unigene32275_All | | 8.6174 | | | 9.2738 | | | 6.6801 | | 0.105908 | | 0.473291 | | 0.367383 |
| Unigene42398_All | | 31.4392 | | | 34.397 | | | 61.3734 | | 0.129718 | | -0.83533 | | -0.96505 |
| Unigene1150_All | | 4.3537 | | | 5.049 | | | 4.2451 | | 0.213756 | | 0.250199 | | 0.036443 |
| Unigene3431_All | | 18.8036 | | | 21.9488 | | | 38.8 | | 0.223133 | | -0.82191 | | -1.04505 |
| Unigene36737_All | | 11.7551 | | | 13.8217 | | | 17.8537 | | 0.233648 | | -0.36929 | | -0.60294 |
| Unigene41477_All | | 3.0428 | | | 3.6037 | | | 4.0911 | | 0.244079 | | -0.18301 | | -0.42709 |
| Unigene1532_All | | 150.7203 | | | 179.0815 | | | 199.814 | | 0.248743 | | -0.15804 | | -0.40678 |
| Unigene26465_All | | 84.0835 | | | 100.5396 | | | 209.239 | | 0.257869 | | -1.05739 | | -1.31526 |
| Unigene38120_All | | 14.7735 | | | 17.7639 | | | 16.1859 | | 0.265937 | | 0.134211 | | -0.13173 |
| Unigene42820_All | | 11.7979 | | | 14.3181 | | | 43.9994 | | 0.27931 | | -1.61964 | | -1.89895 |
| Unigene1745_All | | 21.806 | | | 26.6509 | | | 17.4172 | | 0.289459 | | 0.613672 | | 0.324212 |
| Unigene10610_All | | 1.9933 | | | 2.5125 | | | 1.4775 | | 0.333965 | | 0.765965 | | 0.432001 |
| Unigene34566_All | | 2.7784 | | | 3.537 | | | 1.6165 | | 0.348272 | | 1.129653 | | 0.781381 |
| Unigene42931_All | | 3.3121 | | | 4.2298 | | | 4.5691 | | 0.352843 | | -0.11132 | | -0.46416 |
| Unigene23475_All | | 2.7648 | | | 3.5696 | | | 6.4966 | | 0.368587 | | -0.86392 | | -1.23251 |
| Unigene1746_All | | 4.0309 | | | 5.2471 | | | 1.3709 | | 0.380418 | | 1.936397 | | 1.555979 |
| Unigene18348_All | | 5.1074 | | | 6.6608 | | | 4.8474 | | 0.383106 | | 0.458484 | | 0.075378 |
| Unigene19341_All | | 1.5606 | | | 2.0682 | | | 4.6463 | | 0.406275 | | -1.16771 | | -1.57398 |
| Unigene38035_All | | 38.0082 | | | 50.4323 | | | 47.2973 | | 0.408037 | | 0.09259 | | -0.31545 |
| Unigene27666_All | | 7.5923 | | | 10.1414 | | | 13.4855 | | 0.417648 | | -0.41115 | | -0.8288 |
| Unigene22563_All | | 3.7188 | | | 5.1358 | | | 5.6556 | | 0.465752 | | -0.13909 | | -0.60484 |
| Unigene6478_All | | 1.1313 | | | 1.589 | | | 1.484 | | 0.490138 | | 0.098628 | | -0.39151 |
| Unigene3070_All | | 3.046 | | | 4.3799 | | | 2.8688 | | 0.523982 | | 0.610451 | | 0.086469 |
| Unigene20667_All | | 3.4113 | | | 4.9575 | | | 3.1612 | | 0.539291 | | 0.64914 | | 0.109849 |
| Unigene35501_All | | 0.3644 | | | 0.5307 | | | 0.4337 | | 0.542374 | | 0.291199 | | -0.25117 |
| Unigene79896_All | | 0.1 | | | 0.1459 | | | 1.5667 | | 0.54498 | | -3.42468 | | -3.96966 |
| Unigene19418_All | | 1.4515 | | | 2.227 | | | 4.9548 | | 0.617557 | | -1.15373 | | -1.77128 |
| Unigene57925_All | | 1.6648 | | | 2.5676 | | | 3.9967 | | 0.625072 | | -0.63839 | | -1.26346 |
| Unigene17935_All | | 0.9395 | | | 1.4743 | | | 0.7843 | | 0.650065 | | 0.910553 | | 0.260488 |
| Unigene1181_All | | 3.3236 | | | 5.4002 | | | 4.6648 | | 0.700266 | | 0.211198 | | -0.48907 |
| Unigene7169_All | | 2.4784 | | | 4.0612 | | | 2.9501 | | 0.712497 | | 0.461142 | | -0.25135 |
| Unigene70092_All | | 0.1338 | | | 0.2193 | | | 1.3654 | | 0.712828 | | -2.63835 | | -3.35117 |
| Unigene13699_All | | 1.57 | | | 2.5959 | | | 2.7275 | | 0.72547 | | -0.07134 | | -0.79681 |
| Unigene15509_All | | 0.6694 | | | 1.1135 | | | 1.3815 | | 0.734161 | | -0.31113 | | -1.0453 |
| Unigene60747_All | | 4.8582 | | | 8.1192 | | | 1.7044 | | 0.740916 | | 2.252074 | | 1.511158 |
| Unigene1534_All | | 111.712 | | | 190.0306 | | | 231.483 | | 0.766448 | | -0.28468 | | -1.05112 |
| Unigene31315_All | | 3.1771 | | | 5.4614 | | | 1.0487 | | 0.78156 | | 2.380669 | | 1.599108 |
| Unigene26399_All | | 63.743 | | | 110.7344 | | | 100.159 | | 0.796765 | | 0.144811 | | -0.65195 |
| Unigene22627_All | | 3.0646 | | | 5.3632 | | | 8.3483 | | 0.807395 | | -0.63839 | | -1.44578 |
| Unigene35502_All | | 3.3979 | | | 6.058 | | | 3.3836 | | 0.834198 | | 0.840283 | | 0.006084 |
| Unigene48889_All | | 0.3915 | | | 0.6998 | | | 0.0726 | | 0.83793 | | 3.268901 | | 2.430971 |
| Unigene21046_All | | 3.4635 | | | 6.2963 | | | 3.9203 | | 0.862274 | | 0.68354 | | -0.17873 |
| Unigene32700_All | | 0.2411 | | | 0.4516 | | | 0.0351 | | 0.905414 | | 3.685503 | | 2.780089 |
| Unigene22383_All | | 92.8165 | | | 176.807 | | | 159.62 | | 0.929722 | | 0.147538 | | -0.78218 |
| Unigene19417_All | | 2.1024 | | | 4.0551 | | | 2.603 | | 0.9477 | | 0.639562 | | -0.30814 |
| Unigene41914_All | | 5.3055 | | | 10.482 | | | 5.1828 | | 0.982353 | | 1.01611 | | 0.033757 |
| Unigene38433_All | | 39.2016 | | | 78.0806 | | | 72.2127 | | 0.994052 | | 0.112712 | | -0.88134 |
| Unigene42418_All | | 19.9318 | | | 39.7043 | | | 31.3263 | | 0.994223 | | 0.341921 | | -0.6523 |
| Unigene4497_All | | 13.7917 | | | 29.3955 | | | 42.4209 | | 1.091795 | | -0.52918 | | -1.62097 |
| Unigene50607_All | | 0.55 | | | 1.1744 | | | 0.5101 | | 1.09442 | | 1.203072 | | 0.108652 |
| Unigene4865_All | | 0.587 | | | 1.2578 | | | 2.5223 | | 1.09947 | | -1.00384 | | -2.10331 |
| Unigene41529_All | | 23.8008 | | | 52.1117 | | | 35.1446 | | 1.130597 | | 0.568304 | | -0.56229 |
| Unigene29428_All | | 1.181 | | | 2.6611 | | | 1.7071 | | 1.172014 | | 0.640475 | | -0.53154 |
| Unigene11869_All | | 2.346 | | | 5.5498 | | | 1.1555 | | 1.242233 | | 2.263919 | | 1.021686 |
| Unigene1533_All | | 3.8039 | | | 9.0383 | | | 8.1017 | | 1.248572 | | 0.157827 | | -1.09075 |
| Unigene16890_All | | 6.2236 | | | 15.0215 | | | 7.1368 | | 1.271208 | | 1.07368 | | -0.19753 |
| Unigene1180_All | | 1.0368 | | | 2.5485 | | | 0.3702 | | 1.297511 | | 2.783272 | | 1.485761 |
| Unigene80022_All | | 1.9279 | | | 4.8845 | | | 9.7515 | | 1.341181 | | -0.99741 | | -2.33859 |
| Unigene80299_All | | 0.1 | | | 0.2931 | | | 0.9579 | | 1.551393 | | -1.70848 | | -3.25988 |
| Unigene18862_All | | 4.3227 | | | 13.8374 | | | 5.2104 | | 1.678568 | | 1.409107 | | -0.26946 |
| Unigene73870_All | | 0.1283 | | | 0.4205 | | | 1.7673 | | 1.712585 | | -2.07137 | | -3.78395 |
| Unigene4496_All | | 0.2738 | | | 0.8975 | | | 1.2107 | | 1.712789 | | -0.43186 | | -2.14465 |
| Unigene70583_All | | 0.4539 | | | 1.6403 | | | 1.2113 | | 1.853513 | | 0.437403 | | -1.41611 |
| Unigene40073_All | | 0.147 | | | 0.5418 | | | 1.1995 | | 1.881944 | | -1.1466 | | -3.02855 |
| Unigene46429_All | | 1.2247 | | | 4.7494 | | | 0.1249 | | 1.955317 | | 5.2489 | | 3.293583 |
| Unigene34958_All | | 4.5994 | | | 21.2376 | | | 9.7946 | | 2.207103 | | 1.116562 | | -1.09054 |
| Unigene16128_All | | 1.1096 | | | 7.1798 | | | 2.1336 | | 2.693904 | | 1.750654 | | -0.94325 |
| Unigene26816_All | | 42.1542 | | | 285.6709 | | | 79.4294 | | 2.760606 | | 1.846609 | | -0.914 |
| Unigene42558_All | | 11.2168 | | | 80.492 | | | 19.9278 | | 2.843184 | | 2.014063 | | -0.82912 |
| Unigene45765_All | | 0.1788 | | | 1.6741 | | | 0.8078 | | 3.226967 | | 1.051316 | | -2.17565 |
| Unigene30037_All | | 0.1 | | | 1.4928 | | | 2.4897 | | 3.899949 | | -0.73795 | | -4.6379 |
| NAC | | OW_RPKM | | | TW_RPKM | | | NW_RPKM | | T/O | | T/N | | O/N |
| Unigene15438_All | | 5.201 | | | 0.5411 | | | 25.353 | | -3.26482 | | -5.55012 | | -2.2853 |
| Unigene33234_All | | 0.2585 | | | 0.0282 | | | 1.1474 | | -3.1964 | | -5.34653 | | -2.15013 |
| Unigene75669_All | | 0.5871 | | | 0.0802 | | | 1.5724 | | -2.87193 | | -4.29322 | | -1.42129 |
| Unigene22134_All | | 18.6884 | | | 2.7694 | | | 6.5964 | | -2.7545 | | -1.25211 | | 1.502392 |
| Unigene41490_All | | 4.7768 | | | 0.8489 | | | 8.6515 | | -2.49238 | | -3.34928 | | -0.85691 |
| Unigene33685_All | | 52.2662 | | | 11.0219 | | | 22.0006 | | -2.24551 | | -0.99717 | | 1.248335 |
| Unigene42409_All | | 0.1267 | | | 0.0277 | | | 0.0517 | | -2.19346 | | -0.90028 | | 1.29318 |
| Unigene22008_All | | 702.6978 | | | 154.8669 | | | 109.124 | | -2.18188 | | 0.505063 | | 2.686939 |
| Unigene25354_All | | 0.3103 | | | 0.0782 | | | 0.414 | | -1.98842 | | -2.40439 | | -0.41597 |
| Unigene11418_All | | 240.2747 | | | 65.8363 | | | 31.9817 | | -1.86773 | | 1.041637 | | 2.909366 |
| Unigene43402_All | | 0.2205 | | | 0.0688 | | | 0.0643 | | -1.6803 | | 0.09759 | | 1.777888 |
| Unigene35383_All | | 10.319 | | | 3.4044 | | | 8.1287 | | -1.59983 | | -1.25562 | | 0.344207 |
| Unigene70082_All | | 10.1297 | | | 3.4332 | | | 7.5008 | | -1.56097 | | -1.12749 | | 0.433475 |
| Unigene49159_All | | 4.1221 | | | 1.6176 | | | 2.2866 | | -1.34952 | | -0.49935 | | 0.850175 |
| Unigene39342_All | | 16.7718 | | | 6.7072 | | | 19.0218 | | -1.32226 | | -1.50387 | | -0.18162 |
| Unigene33683_All | | 22.6821 | | | 9.2132 | | | 8.9779 | | -1.29978 | | 0.037324 | | 1.337104 |
| Unigene50011_All | | 2.1324 | | | 0.9115 | | | 1.5135 | | -1.22616 | | -0.73157 | | 0.494589 |
| Unigene28959_All | | 0.3192 | | | 0.1395 | | | 0.1845 | | -1.1942 | | -0.40336 | | 0.79084 |
| Unigene41961_All | | 41.9341 | | | 21.4224 | | | 39.6505 | | -0.969 | | -0.88822 | | 0.080785 |
| Unigene22661_All | | 4.889 | | | 2.7034 | | | 3.8489 | | -0.85476 | | -0.50967 | | 0.345093 |
| Unigene19662_All | | 2.3786 | | | 1.3294 | | | 1.3545 | | -0.83934 | | -0.02699 | | 0.812352 |
| Unigene26602_All | | 10.6816 | | | 5.9737 | | | 13.7578 | | -0.83843 | | -1.20355 | | -0.36512 |
| Unigene43403_All | | 1.2465 | | | 0.7427 | | | 0.6648 | | -0.74703 | | 0.159859 | | 0.906891 |
| Unigene53540_All | | 1.5566 | | | 1.0203 | | | 0.7147 | | -0.6094 | | 0.513584 | | 1.122989 |
| Unigene22057_All | | 4.3274 | | | 2.9509 | | | 8.9429 | | -0.55235 | | -1.59959 | | -1.04724 |
| Unigene2377_All | | 39.4233 | | | 29.6836 | | | 26.6401 | | -0.40938 | | 0.156067 | | 0.565449 |
| Unigene35774_All | | 13.4886 | | | 10.6636 | | | 14.6045 | | -0.33905 | | -0.45372 | | -0.11467 |
| Unigene26322_All | | 5.3848 | | | 4.2701 | | | 2.8167 | | -0.33462 | | 0.600264 | | 0.934887 |
| Unigene2081_All | | 137.4793 | | | 109.6385 | | | 133.18 | | -0.32646 | | -0.28062 | | 0.045836 |
| Unigene2080_All | | 81.4637 | | | 65.4574 | | | 81.0342 | | -0.3156 | | -0.30797 | | 0.007626 |
| Unigene34192_All | | 7.3863 | | | 6.1259 | | | 5.5675 | | -0.26993 | | 0.137892 | | 0.407822 |
| Unigene46701_All | | 1.1722 | | | 0.985 | | | 0.736 | | -0.25102 | | 0.420418 | | 0.671441 |
| Unigene4707_All | | 2.592 | | | 2.2871 | | | 4.2042 | | -0.18055 | | -0.87831 | | -0.69777 |
| Unigene35169_All | | 5.8998 | | | 5.2562 | | | 2.9221 | | -0.16665 | | 0.847015 | | 1.01366 |
| Unigene68484_All | | 3.3069 | | | 2.9651 | | | 0.5729 | | -0.1574 | | 2.371726 | | 2.529124 |
| Unigene41278_All | | 28.7442 | | | 26.0791 | | | 46.6955 | | -0.14038 | | -0.84039 | | -0.70001 |
| Unigene43690_All | | 10.8443 | | | 10.1774 | | | 6.538 | | -0.09157 | | 0.638448 | | 0.730016 |
| Unigene36395_All | | 13.7533 | | | 12.9292 | | | 13.559 | | -0.08914 | | -0.06862 | | 0.020527 |
| Unigene43276_All | | 6.2188 | | | 5.9247 | | | 5.7547 | | -0.06989 | | 0.042001 | | 0.111895 |
| Unigene50147_All | | 0.522 | | | 0.5214 | | | 0.1217 | | -0.00166 | | 2.099061 | | 2.100721 |
| Unigene4777_All | | 3.5655 | | | 3.995 | | | 5.9697 | | 0.164091 | | -0.57946 | | -0.74355 |
| Unigene35773_All | | 9.1649 | | | 10.2711 | | | 3.8424 | | 0.1644 | | 1.418511 | | 1.254111 |
| Unigene26722_All | | 173.7794 | | | 198.7826 | | | 260.209 | | 0.193934 | | -0.38848 | | -0.58241 |
| Unigene40575_All | | 1.2035 | | | 1.3987 | | | 2.0117 | | 0.21685 | | -0.52433 | | -0.74118 |
| Unigene2902_All | | 5.4443 | | | 6.399 | | | 5.793 | | 0.2331 | | 0.143536 | | -0.08956 |
| Unigene43507_All | | 5.6774 | | | 6.9715 | | | 2.1105 | | 0.296239 | | 1.723884 | | 1.427646 |
| Unigene1583_All | | 20.333 | | | 26.5074 | | | 16.2839 | | 0.382572 | | 0.702949 | | 0.320377 |
| Unigene42376_All | | 55.4709 | | | 73.5583 | | | 63.2362 | | 0.407157 | | 0.218137 | | -0.18902 |
| Unigene25128_All | | 1.1648 | | | 1.5491 | | | 1.5501 | | 0.411348 | | -0.00093 | | -0.41228 |
| Unigene10635_All | | 15.5337 | | | 20.7547 | | | 18.9948 | | 0.418037 | | 0.127834 | | -0.2902 |
| Unigene34191_All | | 1.4498 | | | 2.0193 | | | 1.0724 | | 0.478001 | | 0.913012 | | 0.435011 |
| Unigene38266_All | | 14.6949 | | | 20.9374 | | | 13.4609 | | 0.510767 | | 0.637307 | | 0.126541 |
| Unigene26647_All | | 2.2126 | | | 3.5228 | | | 2.2574 | | 0.67098 | | 0.64206 | | -0.02892 |
| Unigene8542_All | | 1.2497 | | | 2.0478 | | | 1.6575 | | 0.712493 | | 0.305066 | | -0.40743 |
| Unigene33233_All | | 1.3396 | | | 2.289 | | | 1.1741 | | 0.772915 | | 0.963162 | | 0.190247 |
| Unigene6528_All | | 3.1463 | | | 5.4503 | | | 2.4305 | | 0.792679 | | 1.165083 | | 0.372403 |
| Unigene21130_All | | 8.9893 | | | 15.602 | | | 14.2935 | | 0.79545 | | 0.126372 | | -0.66908 |
| Unigene3402_All | | 10.3597 | | | 18.5824 | | | 4.6712 | | 0.842955 | | 1.992072 | | 1.149117 |
| Unigene17350_All | | 0.4121 | | | 0.7744 | | | 0.2859 | | 0.910084 | | 1.437568 | | 0.527484 |
| Unigene16889_All | | 2.4516 | | | 4.626 | | | 4.2636 | | 0.916042 | | 0.117693 | | -0.79835 |
| Unigene18594_All | | 0.9925 | | | 1.8793 | | | 0.405 | | 0.921056 | | 2.214202 | | 1.293145 |
| Unigene26149_All | | 19.4123 | | | 36.9674 | | | 20.7873 | | 0.929283 | | 0.830551 | | -0.09873 |
| Unigene4473_All | | 1.352 | | | 2.5847 | | | 2.8451 | | 0.934902 | | -0.13848 | | -1.07338 |
| Unigene30536_All | | 1.0461 | | | 2.0258 | | | 1.5039 | | 0.953471 | | 0.429783 | | -0.52369 |
| Unigene3883_All | | 15.4171 | | | 30.8568 | | | 24.244 | | 1.001057 | | 0.347961 | | -0.6531 |
| Unigene41345_All | | 6.1455 | | | 12.4015 | | | 8.4908 | | 1.012912 | | 0.546542 | | -0.46637 |
| Unigene50196_All | | 12.737 | | | 27.0407 | | | 19.9976 | | 1.086107 | | 0.435306 | | -0.6508 |
| Unigene27679_All | | 4.3131 | | | 9.4684 | | | 2.9337 | | 1.134395 | | 1.690399 | | 0.556004 |
| Unigene60616_All | | 1.0605 | | | 2.5139 | | | 1.8987 | | 1.245183 | | 0.404915 | | -0.84027 |
| Unigene5392_All | | 0.9946 | | | 2.4589 | | | 3.0976 | | 1.305825 | | -0.33314 | | -1.63896 |
| Unigene35175_All | | 1.2214 | | | 3.5264 | | | 1.8396 | | 1.52966 | | 0.938804 | | -0.59086 |
| Unigene10343_All | | 2.3781 | | | 6.8866 | | | 1.2132 | | 1.533983 | | 2.504974 | | 0.970992 |
| Unigene4429_All | | 1.1544 | | | 3.4231 | | | 0.8974 | | 1.56816 | | 1.93148 | | 0.36332 |
| Unigene56064_All | | 0.7783 | | | 2.9286 | | | 2.2058 | | 1.911813 | | 0.408909 | | -1.5029 |
| Unigene6281_All | | 5.7476 | | | 22.4953 | | | 10.3662 | | 1.968592 | | 1.117736 | | -0.85086 |
| Unigene4428_All | | 0.3097 | | | 1.2405 | | | 2.3698 | | 2.001978 | | -0.93384 | | -2.93582 |
| Unigene18595_All | | 1.0155 | | | 4.9458 | | | 1.8994 | | 2.284014 | | 1.38066 | | -0.90335 |
| Unigene58420_All | | 0.1696 | | | 1.4209 | | | 1.2693 | | 3.066597 | | 0.162772 | | -2.90383 |
| Unigene80073_All | | 0.8066 | | | 7.8325 | | | 2.1946 | | 3.279548 | | 1.835515 | | -1.44403 |
| Unigene33462_All | | 1.6717 | | | 18.0037 | | | 4.662 | | 3.428906 | | 1.949273 | | -1.47963 |
| Unigene33461_All | | 0.1334 | | | 2.4293 | | | 1.0663 | | 4.18671 | | 1.187927 | | -2.99878 |
| Unigene29977_All | | 0.0682 | | | 1.3043 | | | 0.5221 | | 4.25736 | | 1.320878 | | -2.93648 |
| Unigene29973_All | | 0.17 | | | 3.3733 | | | 1.7631 | | 4.310554 | | 0.936046 | | -3.37451 |
| Auxin relate | | TW_RPKM | | | NW_RPKM | | | OW_RPKM | | T/O | | T/N | | O/N |
| Unigene67286_All | | 1.5923 | | | 0.048 | | | 0.1411 | | -5.05193 | | -1.55561 | | 3.496322 |
| Unigene479_All | | 0.9488 | | | 0.0306 | | | 0.4942 | | -4.9545 | | -4.01349 | | 0.941009 |
| Unigene37202_All | | 8.0496 | | | 0.3629 | | | 1.4229 | | -4.47127 | | -1.97119 | | 2.500083 |
| Unigene477_All | | 1.8331 | | | 0.1 | | | 1.6081 | | -4.19621 | | -4.00729 | | 0.188928 |
| Unigene24018_All | | 7.4784 | | | 0.4365 | | | 0.8022 | | -4.09868 | | -0.87798 | | 3.220696 |
| Unigene67542_All | | 7.9828 | | | 0.4709 | | | 5.3843 | | -4.0834 | | -3.51527 | | 0.568136 |
| Unigene57336_All | | 0.7919 | | | 0.0493 | | | 0.29 | | -4.00566 | | -2.55639 | | 1.449265 |
| Unigene57915_All | | 1.5136 | | | 0.1 | | | 0.1066 | | -3.91991 | | -0.09221 | | 3.827705 |
| Unigene67376_All | | 9.32 | | | 0.8511 | | | 3.9813 | | -3.45293 | | -2.22584 | | 1.22709 |
| Unigene59418_All | | 1.0507 | | | 0.1 | | | 0.541 | | -3.39328 | | -2.43563 | | 0.95765 |
| Unigene57914_All | | 0.8128 | | | 0.1 | | | 0.1395 | | -3.0229 | | -0.48027 | | 2.542635 |
| Unigene17017_All | | 4.5284 | | | 0.5999 | | | 2.4401 | | -2.91621 | | -2.02415 | | 0.892061 |
| Unigene1362_All | | 1.9704 | | | 0.2706 | | | 1.1406 | | -2.86425 | | -2.07556 | | 0.788696 |
| Unigene32111_All | | 18.1488 | | | 2.6 | | | 17.544 | | -2.80329 | | -2.75442 | | 0.048872 |
| Unigene18949_All | | 4.8655 | | | 0.7128 | | | 1.1935 | | -2.77102 | | -0.74363 | | 2.02739 |
| Unigene68314_All | | 0.654 | | | 0.1 | | | 0.1 | | -2.70929 | | 0 | | 2.709291 |
| Unigene67461_All | | 0.2678 | | | 0.0417 | | | 0.01 | | -2.68304 | | 2.060047 | | 4.743084 |
| Unigene62150_All | | 0.1971 | | | 0.0307 | | | 0.1804 | | -2.68262 | | -2.55489 | | 0.127728 |
| Unigene33753_All | | 4.2288 | | | 0.7103 | | | 1.7962 | | -2.57375 | | -1.33845 | | 1.2353 |
| Unigene42849_All | | 141.0172 | | | 26.2798 | | | 40.973 | | -2.42384 | | -0.64073 | | 1.783115 |
| Unigene62767_All | | 0.5355 | | | 0.1 | | | 0.3268 | | -2.42089 | | -1.70841 | | 0.712479 |
| Unigene11558_All | | 5.8385 | | | 1.107 | | | 0.1 | | -2.39894 | | 3.468583 | | 5.867526 |
| Unigene1678_All | | 3.3054 | | | 0.8479 | | | 1.2572 | | -1.96286 | | -0.56825 | | 1.394611 |
| Unigene50726_All | | 0.6672 | | | 0.178 | | | 0.8724 | | -1.90624 | | -2.29311 | | -0.38687 |
| Unigene61552_All | | 0.7514 | | | 0.2005 | | | 0.1 | | -1.90598 | | 1.003602 | | 2.909581 |
| Unigene55684_All | | 2.5028 | | | 0.6906 | | | 2.226 | | -1.85762 | | -1.68853 | | 0.169089 |
| Unigene46844_All | | 4.4633 | | | 1.2443 | | | 1.2806 | | -1.84278 | | -0.04149 | | 1.801291 |
| Unigene22308_All | | 54.1193 | | | 15.5408 | | | 54.531 | | -1.80008 | | -1.81102 | | -0.01093 |
| Unigene57272_All | | 0.2339 | | | 0.0728 | | | 0.1666 | | -1.68388 | | -1.19438 | | 0.489503 |
| Unigene54701_All | | 0.3385 | | | 0.1054 | | | 0.0689 | | -1.68328 | | 0.613299 | | 2.29658 |
| Unigene52625_All | | 0.321 | | | 0.1 | | | 0.7835 | | -1.68257 | | -2.96993 | | -1.28736 |
| Unigene7851_All | | 5.6835 | | | 1.904 | | | 3.0537 | | -1.57775 | | -0.68152 | | 0.896221 |
| Unigene10535_All | | 1.267 | | | 0.426 | | | 2.9691 | | -1.57249 | | -2.8011 | | -1.22861 |
| Unigene26278_All | | 11.1986 | | | 4.1107 | | | 4.4351 | | -1.44586 | | -0.10958 | | 1.33628 |
| Unigene39784_All | | 0.4986 | | | 0.1863 | | | 0.4108 | | -1.42026 | | -1.14081 | | 0.279447 |
| Unigene37298_All | | 9.4745 | | | 3.5793 | | | 6.4803 | | -1.40437 | | -0.85638 | | 0.547989 |
| Unigene67460_All | | 1.1662 | | | 0.4456 | | | 0.558 | | -1.38799 | | -0.32452 | | 1.063478 |
| Unigene41951_All | | 2720.2 | | | 1080.875 | | | 1521.9 | | -1.33151 | | -0.49372 | | 0.837794 |
| Unigene64890_All | | 1.3896 | | | 0.5678 | | | 0.0795 | | -1.29121 | | 2.836356 | | 4.127571 |
| Unigene15661_All | | 2.9704 | | | 1.2377 | | | 0.9203 | | -1.263 | | 0.427486 | | 1.690481 |
| Unigene3783_All | | 1.9066 | | | 0.8233 | | | 0.2064 | | -1.21151 | | 1.995975 | | 3.207487 |
| Unigene10366_All | | 7.7059 | | | 3.3383 | | | 4.2706 | | -1.20685 | | -0.35533 | | 0.851525 |
| Unigene3146_All | | 12.2968 | | | 5.8242 | | | 4.6149 | | -1.07815 | | 0.335761 | | 1.413912 |
| Unigene21168_All | | 3.6643 | | | 1.7769 | | | 4.5798 | | -1.04418 | | -1.36592 | | -0.32175 |
| Unigene3093_All | | 39.4011 | | | 19.265 | | | 47.543 | | -1.03225 | | -1.30325 | | -0.27099 |
| Unigene18772_All | | 2.5167 | | | 1.2751 | | | 2.1825 | | -0.98092 | | -0.77537 | | 0.205552 |
| Unigene18593_All | | 8.4376 | | | 4.5643 | | | 6.0604 | | -0.88644 | | -0.40902 | | 0.47742 |
| Unigene47866_All | | 2.9343 | | | 1.5913 | | | 2.8593 | | -0.88281 | | -0.84546 | | 0.037354 |
| Unigene47228_All | | 11.4646 | | | 6.7695 | | | 3.7294 | | -0.76006 | | 0.860106 | | 1.620171 |
| Unigene22268_All | | 49.6949 | | | 29.3969 | | | 22.936 | | -0.75743 | | 0.358075 | | 1.115509 |
| Unigene42115_All | | 0.5486 | | | 0.3274 | | | 0.3348 | | -0.7447 | | -0.03225 | | 0.712455 |
| Unigene33833_All | | 2.8251 | | | 1.7381 | | | 3.6533 | | -0.70079 | | -1.07169 | | -0.3709 |
| Unigene68055_All | | 6.1691 | | | 4.0038 | | | 8.8056 | | -0.62369 | | -1.13705 | | -0.51336 |
| Unigene5380_All | | 7.5454 | | | 5.2623 | | | 2.0558 | | -0.5199 | | 1.355994 | | 1.875897 |
| Unigene26380_All | | 1.8112 | | | 1.2901 | | | 1.3207 | | -0.48946 | | -0.03382 | | 0.455643 |
| Unigene19302_All | | 56.5627 | | | 40.6247 | | | 41.188 | | -0.47749 | | -0.01987 | | 0.457627 |
| Unigene27444_All | | 2.142 | | | 1.6005 | | | 4.1503 | | -0.42044 | | -1.37469 | | -0.95426 |
| Unigene33834_All | | 12.4601 | | | 9.3309 | | | 18.014 | | -0.41723 | | -0.94901 | | -0.53178 |
| Unigene15985_All | | 1.9817 | | | 1.4873 | | | 0.6803 | | -0.41404 | | 1.128453 | | 1.542496 |
| Unigene25173_All | | 4.6539 | | | 3.5045 | | | 1.1375 | | -0.40923 | | 1.623342 | | 2.032574 |
| Unigene34091_All | | 2.5678 | | | 1.9558 | | | 1.392 | | -0.39277 | | 0.4906 | | 0.883374 |
| Unigene34020_All | | 94.8585 | | | 72.8317 | | | 87.197 | | -0.38121 | | -0.25971 | | 0.121504 |
| Unigene9853_All | | 57.2829 | | | 44.4007 | | | 57.797 | | -0.36752 | | -0.38041 | | -0.01289 |
| Unigene1679_All | | 20.943 | | | 16.6814 | | | 15.789 | | -0.32823 | | 0.079348 | | 0.407576 |
| Unigene32977_All | | 2.5484 | | | 2.0541 | | | 0.9427 | | -0.31109 | | 1.123636 | | 1.434721 |
| Unigene25897_All | | 53.7694 | | | 43.8969 | | | 59.773 | | -0.29267 | | -0.44538 | | -0.15271 |
| Unigene22233_All | | 51.1224 | | | 42.0728 | | | 157.92 | | -0.28107 | | -1.90819 | | -1.62712 |
| Unigene27206_All | | 6.2039 | | | 5.1057 | | | 6.199 | | -0.28107 | | -0.27993 | | 0.00114 |
| Unigene26862_All | | 402.7405 | | | 333.86 | | | 277.3 | | -0.27061 | | 0.267799 | | 0.538406 |
| Unigene41665_All | | 3.0694 | | | 2.5649 | | | 2.5878 | | -0.25905 | | -0.01282 | | 0.246231 |
| Unigene10212_All | | 11.9879 | | | 10.1105 | | | 11.605 | | -0.24572 | | -0.19885 | | 0.04687 |
| Unigene2522_All | | 4.156 | | | 3.5416 | | | 3.927 | | -0.23079 | | -0.14903 | | 0.081768 |
| Unigene43720_All | | 18.2155 | | | 15.7706 | | | 13.347 | | -0.20793 | | 0.240679 | | 0.448608 |
| Unigene10401_All | | 62.6585 | | | 55.0253 | | | 73.467 | | -0.18742 | | -0.417 | | -0.22959 |
| Unigene935_All | | 283.9327 | | | 255.4066 | | | 164.74 | | -0.15275 | | 0.632615 | | 0.785369 |
| Unigene36788_All | | 20.2803 | | | 18.3043 | | | 19.419 | | -0.1479 | | -0.08525 | | 0.062647 |
| Unigene3848_All | | 3.9146 | | | 3.55 | | | 1.3243 | | -0.14105 | | 1.422589 | | 1.563635 |
| Unigene26199_All | | 28.819 | | | 26.574 | | | 23.125 | | -0.117 | | 0.200581 | | 0.317586 |
| Unigene862_All | | 18.0089 | | | 16.7938 | | | 13.842 | | -0.10078 | | 0.278887 | | 0.379668 |
| Unigene50225_All | | 0.2795 | | | 0.261 | | | 0.5373 | | -0.0988 | | -1.04168 | | -0.94288 |
| Unigene35328_All | | 0.3058 | | | 0.2856 | | | 0.6531 | | -0.09859 | | -1.19331 | | -1.09472 |
| Unigene25062_All | | 66.0013 | | | 64.3985 | | | 71.151 | | -0.03547 | | -0.14386 | | -0.10839 |
| Unigene3355_All | | 1.8254 | | | 1.8266 | | | 1.3606 | | 0.000948 | | 0.424918 | | 0.42397 |
| Unigene10648_All | | 3.9151 | | | 3.9519 | | | 3.6924 | | 0.013497 | | 0.097988 | | 0.08449 |
| Unigene34019_All | | 97.8058 | | | 99.7988 | | | 88.788 | | 0.029102 | | 0.168663 | | 0.13956 |
| Unigene41123_All | | 11.1923 | | | 11.7524 | | | 13.783 | | 0.070449 | | -0.22992 | | -0.30037 |
| Unigene29740_All | | 1.4759 | | | 1.5646 | | | 0.5842 | | 0.084199 | | 1.42126 | | 1.337061 |
| Unigene42204_All | | 6.6841 | | | 7.0946 | | | 4.7673 | | 0.085988 | | 0.573549 | | 0.487561 |
| Unigene58555_All | | 0.6499 | | | 0.6937 | | | 0.17 | | 0.094094 | | 2.028777 | | 1.934683 |
| Unigene3147_All | | 0.1673 | | | 0.1786 | | | 0.0438 | | 0.094295 | | 2.027729 | | 1.933435 |
| Unigene25967_All | | 34.1167 | | | 36.6111 | | | 55.384 | | 0.101803 | | -0.59718 | | -0.69898 |
| Unigene72120_All | | 0.6998 | | | 0.7662 | | | 0.4197 | | 0.130778 | | 0.868363 | | 0.737584 |
| Unigene35329_All | | 2.3626 | | | 2.5927 | | | 1.0273 | | 0.13408 | | 1.335598 | | 1.201518 |
| Unigene36923_All | | 208.8755 | | | 236.4483 | | | 202.12 | | 0.178881 | | 0.226345 | | 0.047463 |
| Unigene42050_All | | 36.4963 | | | 42.0265 | | | 37.05 | | 0.203549 | | 0.18183 | | -0.02172 |
| Unigene50647_All | | 0.5128 | | | 0.5987 | | | 0.3521 | | 0.223437 | | 0.765848 | | 0.542411 |
| Unigene68107_All | | 16.9912 | | | 19.9187 | | | 13.008 | | 0.229336 | | 0.614724 | | 0.385389 |
| Unigene11310_All | | 7.4267 | | | 8.7739 | | | 6.7983 | | 0.240497 | | 0.368044 | | 0.127547 |
| Unigene53567_All | | 0.2751 | | | 0.3304 | | | 2.6265 | | 0.264258 | | -2.99086 | | -3.25511 |
| Unigene20908_All | | 1.3219 | | | 1.5977 | | | 1.2101 | | 0.273383 | | 0.40087 | | 0.127487 |
| Unigene10742_All | | 0.5457 | | | 0.6678 | | | 0.6545 | | 0.291308 | | 0.029023 | | -0.26229 |
| Unigene18096_All | | 35.442 | | | 43.7535 | | | 26.385 | | 0.303938 | | 0.729708 | | 0.425769 |
| Unigene58526_All | | 2.6547 | | | 3.2896 | | | 2.679 | | 0.309363 | | 0.296218 | | -0.01315 |
| Unigene21102_All | | 13.577 | | | 16.8334 | | | 10.231 | | 0.310162 | | 0.718422 | | 0.40826 |
| Unigene4598_All | | 2.1769 | | | 2.7019 | | | 0.8128 | | 0.311699 | | 1.733002 | | 1.421303 |
| Unigene18950_All | | 6.3804 | | | 7.9875 | | | 3.4617 | | 0.324097 | | 1.206263 | | 0.882166 |
| Unigene23227_All | | 2.4567 | | | 3.1288 | | | 0.8178 | | 0.348888 | | 1.935789 | | 1.586902 |
| Unigene648_All | | 13.1077 | | | 16.7252 | | | 16.327 | | 0.351609 | | 0.034773 | | -0.31684 |
| Unigene67285_All | | 1.7221 | | | 2.2085 | | | 1.9999 | | 0.358898 | | 0.143139 | | -0.21576 |
| Unigene33414_All | | 10.4056 | | | 13.5732 | | | 5.7706 | | 0.383401 | | 1.233968 | | 0.850567 |
| Unigene35932_All | | 61.5282 | | | 81.0379 | | | 68.735 | | 0.397349 | | 0.23756 | | -0.15979 |
| Unigene42239_All | | 56.1652 | | | 73.9897 | | | 59.711 | | 0.397648 | | 0.309333 | | -0.08832 |
| Unigene10376_All | | 44.8373 | | | 59.8278 | | | 47.481 | | 0.416117 | | 0.333454 | | -0.08266 |
| Unigene19885_All | | 31.5484 | | | 42.2996 | | | 32.079 | | 0.423077 | | 0.398997 | | -0.02408 |
| Unigene20390_All | | 5.967 | | | 8.0195 | | | 4.2187 | | 0.426507 | | 0.926714 | | 0.500207 |
| Unigene43144_All | | 3.1898 | | | 4.3245 | | | 2.0722 | | 0.439067 | | 1.06137 | | 0.622303 |
| Unigene35189_All | | 5.9945 | | | 8.1922 | | | 7.0063 | | 0.450612 | | 0.225598 | | -0.22501 |
| Unigene79233_All | | 0.3119 | | | 0.4272 | | | 0.1523 | | 0.453828 | | 1.487996 | | 1.034168 |
| Unigene14859_All | | 1.1567 | | | 1.5844 | | | 0.1 | | 0.453922 | | 3.985865 | | 3.531943 |
| Unigene48417_All | | 0.5031 | | | 0.7048 | | | 0.6908 | | 0.486369 | | 0.028946 | | -0.45742 |
| Unigene51808_All | | 0.5225 | | | 0.732 | | | 0.4783 | | 0.486413 | | 0.613928 | | 0.127515 |
| Unigene41350_All | | 17.8181 | | | 25.2495 | | | 17.326 | | 0.502911 | | 0.54335 | | 0.040438 |
| Unigene55372_All | | 2.1117 | | | 3.0077 | | | 0.8215 | | 0.510256 | | 1.872328 | | 1.362072 |
| Unigene18999_All | | 2.6877 | | | 3.8813 | | | 2.7601 | | 0.530168 | | 0.491819 | | -0.03835 |
| Unigene9159_All | | 19.3855 | | | 28.5481 | | | 13.367 | | 0.558417 | | 1.09473 | | 0.536313 |
| Unigene30039_All | | 1.9252 | | | 2.8924 | | | 2.222 | | 0.587259 | | 0.380408 | | -0.20685 |
| Unigene3822_All | | 10.309 | | | 16.0367 | | | 8.5288 | | 0.637473 | | 0.910963 | | 0.27349 |
| Unigene9026_All | | 16.3082 | | | 25.6718 | | | 18.611 | | 0.654587 | | 0.464021 | | -0.19057 |
| Unigene3053_All | | 3.672 | | | 5.8102 | | | 3.6298 | | 0.662022 | | 0.678698 | | 0.016676 |
| Unigene18773_All | | 0.9777 | | | 1.5505 | | | 1.2495 | | 0.66527 | | 0.311383 | | -0.35389 |
| Unigene3356_All | | 0.3361 | | | 0.538 | | | 0.3076 | | 0.678716 | | 0.806551 | | 0.127835 |
| Unigene10791_All | | 9.9068 | | | 15.9012 | | | 1.7773 | | 0.682645 | | 3.161377 | | 2.478732 |
| Unigene35442_All | | 1.8917 | | | 3.1103 | | | 1.4333 | | 0.71737 | | 1.117713 | | 0.400343 |
| Unigene25246_All | | 31.1268 | | | 51.9377 | | | 64.149 | | 0.738625 | | -0.30464 | | -1.04327 |
| Unigene17018_All | | 5.8173 | | | 9.8713 | | | 8.0501 | | 0.76289 | | 0.294233 | | -0.46866 |
| Unigene42261_All | | 36.0507 | | | 62.1554 | | | 51.251 | | 0.785852 | | 0.278311 | | -0.50754 |
| Unigene79863_All | | 0.6286 | | | 1.0959 | | | 0.2302 | | 0.801902 | | 2.251156 | | 1.449254 |
| Unigene71098_All | | 0.1 | | | 0.1752 | | | 0.1 | | 0.809003 | | 0.809003 | | 0 |
| Unigene70758_All | | 0.9565 | | | 1.6874 | | | 0.1946 | | 0.818965 | | 3.116218 | | 2.297253 |
| Unigene18771_All | | 1.726 | | | 3.0629 | | | 2.5082 | | 0.827466 | | 0.288246 | | -0.53922 |
| Unigene48620_All | | 8.2798 | | | 14.9195 | | | 7.744 | | 0.849531 | | 0.946048 | | 0.096517 |
| Unigene35435_All | | 1.8807 | | | 3.4229 | | | 1.5891 | | 0.863949 | | 1.107009 | | 0.24306 |
| Unigene48397_All | | 0.5016 | | | 0.9369 | | | 0.4974 | | 0.901358 | | 0.913489 | | 0.012131 |
| Unigene9543_All | | 5.2096 | | | 9.8245 | | | 5.7775 | | 0.915211 | | 0.765939 | | -0.14927 |
| Unigene26562_All | | 37.5972 | | | 74.4008 | | | 31.415 | | 0.984693 | | 1.243878 | | 0.259185 |
| Unigene27646_All | | 1.462 | | | 2.9583 | | | 2.7657 | | 1.016825 | | 0.097124 | | -0.9197 |
| Unigene12086_All | | 9.7212 | | | 20.2788 | | | 11.388 | | 1.060766 | | 0.832407 | | -0.22836 |
| Unigene25319_All | | 0.1422 | | | 0.2988 | | | 0.0976 | | 1.071259 | | 1.614227 | | 0.542968 |
| Unigene36729_All | | 148.5771 | | | 320.6712 | | | 275.45 | | 1.109883 | | 0.219282 | | -0.8906 |
| Unigene3768_All | | 0.2928 | | | 0.6381 | | | 0.5361 | | 1.123867 | | 0.25128 | | -0.87259 |
| Unigene49722_All | | 0.8237 | | | 1.7951 | | | 4.1891 | | 1.123873 | | -1.22258 | | -2.34645 |
| Unigene10361_All | | 85.8982 | | | 201.5868 | | | 249.03 | | 1.230701 | | -0.30494 | | -1.53564 |
| Unigene41844_All | | 1.2218 | | | 2.8844 | | | 0.8699 | | 1.239263 | | 1.72935 | | 0.490087 |
| Unigene42404_All | | 26.9663 | | | 64.0863 | | | 59.65 | | 1.248858 | | 0.103496 | | -1.14536 |
| Unigene19614_All | | 0.3389 | | | 0.8177 | | | 2.0165 | | 1.270712 | | -1.30221 | | -2.57292 |
| Unigene25163_All | | 26.5272 | | | 64.7359 | | | 44.876 | | 1.287094 | | 0.528615 | | -0.75848 |
| Unigene43418_All | | 8.7876 | | | 21.5361 | | | 15.907 | | 1.293216 | | 0.437132 | | -0.85608 |
| Unigene36379_All | | 3.9426 | | | 9.8886 | | | 3.8132 | | 1.326619 | | 1.374764 | | 0.048145 |
| Unigene19144_All | | 8.0499 | | | 20.631 | | | 12.834 | | 1.357771 | | 0.684854 | | -0.67292 |
| Unigene21222_All | | 1.9681 | | | 5.093 | | | 4.5039 | | 1.371712 | | 0.177341 | | -1.19437 |
| Unigene74998_All | | 0.1 | | | 0.2668 | | | 0.1 | | 1.415759 | | 1.415759 | | 0 |
| Unigene50233_All | | 0.1 | | | 0.2714 | | | 0.266 | | 1.440421 | | 0.028994 | | -1.41143 |
| Unigene30340_All | | 1.0422 | | | 2.8391 | | | 1.6696 | | 1.445802 | | 0.765931 | | -0.67987 |
| Unigene17019_All | | 3.1935 | | | 8.7451 | | | 5.7021 | | 1.453336 | | 0.616982 | | -0.83635 |
| Unigene10700_All | | 1.9724 | | | 5.5465 | | | 2.983 | | 1.491626 | | 0.894814 | | -0.59681 |
| Unigene36670_All | | 2.9577 | | | 8.3807 | | | 1.4914 | | 1.502595 | | 2.490404 | | 0.987808 |
| Unigene20953_All | | 0.9767 | | | 2.8351 | | | 4.0956 | | 1.537412 | | -0.53068 | | -2.06809 |
| Unigene26408_All | | 0.9421 | | | 2.7996 | | | 1.8619 | | 1.571269 | | 0.588445 | | -0.98282 |
| Unigene2470_All | | 2.4899 | | | 7.6776 | | | 3.3645 | | 1.624568 | | 1.190263 | | -0.4343 |
| Unigene12543_All | | 1.9482 | | | 6.043 | | | 2.4733 | | 1.633123 | | 1.288828 | | -0.3443 |
| Unigene69790_All | | 0.0549 | | | 0.1795 | | | 0.01 | | 1.709106 | | 4.165912 | | 2.456806 |
| Unigene73978_All | | 12.9237 | | | 44.3166 | | | 21.179 | | 1.777828 | | 1.06522 | | -0.71261 |
| Unigene2868_All | | 1.8477 | | | 6.5192 | | | 4.1344 | | 1.818964 | | 0.657017 | | -1.16195 |
| Unigene46482_All | | 2.3704 | | | 8.5665 | | | 3.0189 | | 1.853575 | | 1.504683 | | -0.34889 |
| Unigene9844_All | | 13.0722 | | | 48.0721 | | | 25.734 | | 1.878698 | | 0.90153 | | -0.97717 |
| Unigene79950_All | | 0.1568 | | | 0.5859 | | | 0.1 | | 1.901729 | | 2.550654 | | 0.648926 |
| Unigene3769_All | | 2.2437 | | | 8.861 | | | 5.7508 | | 1.98159 | | 0.623707 | | -1.35788 |
| Unigene39159_All | | 0.6351 | | | 2.5208 | | | 1.308 | | 1.988826 | | 0.946519 | | -1.04231 |
| Unigene41152_All | | 0.4722 | | | 1.8899 | | | 1.3584 | | 2.00084 | | 0.476402 | | -1.52444 |
| Unigene1361_All | | 0.1177 | | | 0.4839 | | | 0.2156 | | 2.039595 | | 1.166352 | | -0.87324 |
| Unigene77935_All | | 0.4001 | | | 1.6816 | | | 0.4578 | | 2.071402 | | 1.877045 | | -0.19436 |
| Unigene861_All | | 4.9311 | | | 22.4448 | | | 6.9443 | | 2.1864 | | 1.69248 | | -0.49392 |
| Unigene36230_All | | 4.459 | | | 20.8051 | | | 6.8709 | | 2.222145 | | 1.598366 | | -0.62378 |
| Unigene1364_All | | 0.0444 | | | 0.2075 | | | 0.122 | | 2.22448 | | 0.76623 | | -1.45825 |
| Unigene24107_All | | 1.0705 | | | 5.0898 | | | 1.5144 | | 2.249324 | | 1.748863 | | -0.50046 |
| Unigene8369_All | | 0.543 | | | 2.7259 | | | 2.4232 | | 2.327709 | | 0.169819 | | -2.15789 |
| Unigene41936_All | | 1.7928 | | | 9.0134 | | | 6.2852 | | 2.329857 | | 0.520113 | | -1.80974 |
| Unigene42729_All | | 75.1467 | | | 395.597 | | | 142.8 | | 2.39625 | | 1.470048 | | -0.9262 |
| Unigene7757_All | | 0.3018 | | | 1.6305 | | | 0.4143 | | 2.43365 | | 1.976567 | | -0.45708 |
| Unigene18094_All | | 2.1258 | | | 12.2079 | | | 3.9723 | | 2.521737 | | 1.619769 | | -0.90197 |
| Unigene19595_All | | 0.4408 | | | 2.6758 | | | 3.3086 | | 2.601774 | | -0.30625 | | -2.90802 |
| Unigene70111_All | | 0.3843 | | | 2.9009 | | | 1.7294 | | 2.916196 | | 0.746229 | | -2.16997 |
| Unigene47112_All | | 0.2663 | | | 2.031 | | | 0.4469 | | 2.931066 | | 2.184166 | | -0.7469 |
| Unigene9564_All | | 6.7032 | | | 51.6275 | | | 27.53 | | 2.945218 | | 0.907135 | | -2.03808 |
| Unigene6143_All | | 0.8077 | | | 6.3252 | | | 2.0475 | | 2.96922 | | 1.627248 | | -1.34197 |
| Unigene5185_All | | 0.4598 | | | 3.6694 | | | 1.4539 | | 2.996466 | | 1.335616 | | -1.66085 |
| Unigene54872_All | | 0.1 | | | 0.8062 | | | 0.1129 | | 3.011138 | | 2.836092 | | -0.17505 |
| Unigene78700_All | | 0.0924 | | | 0.777 | | | 0.0846 | | 3.07195 | | 3.199185 | | 0.127235 |
| Unigene75325_All | | 0.4925 | | | 4.3239 | | | 0.8115 | | 3.134138 | | 2.41367 | | -0.72047 |
| Unigene44595_All | | 0.6257 | | | 5.525 | | | 1.4059 | | 3.142431 | | 1.97448 | | -1.16795 |
| Unigene12340_All | | 0.0802 | | | 0.8236 | | | 1.3944 | | 3.36027 | | -0.75963 | | -4.1199 |
| Unigene3092_All | | 1.167 | | | 15.2762 | | | 5.9414 | | 3.710409 | | 1.362411 | | -2.348 |
| Unigene19594_All | | 0.0771 | | | 1.2963 | | | 1.4823 | | 4.071525 | | -0.19344 | | -4.26496 |
| Unigene73840_All | | 0.307 | | | 7.0241 | | | 1.8265 | | 4.516003 | | 1.943232 | | -2.57277 |
| Unigene75195_All | | 0.1 | | | 2.883 | | | 0.1 | | 4.849499 | | 4.849499 | | 0 |
| Unigene45809_All | | 0.5826 | | | 20.0052 | | | 5.0044 | | 5.101726 | | 1.999106 | | -3.10262 |
| Unigene71650_All | | 0.0934 | | | 4.535 | | | 1.4531 | | 5.601536 | | 1.641969 | | -3.95957 |

All gene families described in the Discussion are listed, including genes that were differentially expressed and those that were not. The differentially expressed genes were identified by |log2Ratio| ≥ 1.
